# Systemic Radical Scavenger Treatment of a Mouse Model of Rett Syndrome: Merits and Limitations of the Vitamin E Derivative Trolox

**DOI:** 10.3389/fncel.2016.00266

**Published:** 2016-11-15

**Authors:** Oliwia A. Janc, Marc A. Hüser, Katharina Dietrich, Belinda Kempkes, Christiane Menzfeld, Swen Hülsmann, Michael Müller

**Affiliations:** ^1^Center for Nanoscale Microscopy and Molecular Physiology of the Brain (CNMPB)Göttingen, Germany; ^2^Zentrum Physiologie und Pathophysiologie, Institut für Neuro- und Sinnesphysiologie, Universitätsmedizin Göttingen, Georg-August-UniversitätGöttingen, Germany; ^3^Klinik für Anästhesiologie, Universitätsmedizin Göttingen, Georg-August-UniversitätGöttingen, Germany

**Keywords:** redox signaling, oxidative stress, reactive oxygen species (ROS), Rett syndrome, antioxidants, preclinical study, disease progression, pharmacotherapy

## Abstract

Rett syndrome (RTT) is a severe neurodevelopmental disorder typically arising from spontaneous mutations in the X-chromosomal methyl-CpG binding protein 2 (*MECP2*) gene. The almost exclusively female Rett patients show an apparently normal development during their first 6–18 months of life. Subsequently, cognitive- and motor-impairment, hand stereotypies, loss of learned skills, epilepsy and irregular breathing manifest. Early mitochondrial impairment and oxidative challenge are considered to facilitate disease progression. Along this line, we recently confirmed *in vitro* that acute treatment with the vitamin E-derivative Trolox dampens neuronal hyperexcitability, reinstates synaptic plasticity, ameliorates cellular redox balance and improves hypoxia tolerance in male MeCP2-deficient (*Mecp2^−/y^*) mouse hippocampus. Pursuing these promising findings, we performed a preclinical study to define the merit of systemic Trolox administration. Blinded, placebo-controlled *in vivo* treatment of male mice started at postnatal day (PD) 10–11 and continued for ~40 days. Compounds (vehicle only, 10 mg/kg or 40 mg/kg Trolox) were injected intraperitoneally every 48 h. Detailed phenotyping revealed that in *Mecp2^−/y^* mice, blood glucose levels, lipid peroxidation, synaptic short-term plasticity, hypoxia tolerance and certain forms of environmental exploration were improved by Trolox. Yet, body weight and size, motor function and the rate and regularity of breathing did not improve. In conclusion, *in vivo* Trolox treatment partially ameliorated a subset of symptoms of the complex Rett phenotype, thereby confirming a partial merit of the vitamin E-derivative based pharmacotherapy. Yet, it also became evident that frequent animal handling and the route of drug administration are critical issues to be optimized in future trials.

## Introduction

Rett syndrome (RTT) is a progressive neurodevelopmental disorder that almost exclusively affects girls with an incidence of ~1/10,000 live female births (Rett, 1966; Hagberg, 1985; Chahrour and Zoghbi, 2007). Spontaneous mutations in the X-chromosomal methyl-CpG binding protein 2 gene (*MECP2*) are present in 95% of classic Rett patients (Amir et al., 1999; Shahbazian and Zoghbi, 2001). RTT is characterized by an apparently normal development for the first 6–18 months of life. This is then followed by a gradual appearance of mental, neurological and physical symptoms. Rett girls often show autism-like behaviors, severe mental impairment, slowed growth, motor dysfunction and seizures. Other symptoms include cardiac abnormalities and pronounced breathing disturbances (Shahbazian and Zoghbi, 2001). Especially the awake state is characterized by phases of irregular breathing with apneas and associated transient drops in arterial O_2_ saturation (Julu et al., 2001; Stettner et al., 2008).

Mitochondrial alterations contribute to cellular redox imbalance, and are therefore expected to be involved in the pathogenesis of RTT (De Felice et al., 2012b; Müller and Can, 2014; Filosa et al., 2015). Typical morphological changes of these organelles include a swollen appearance with vacuolization, granular inclusions and membranous changes, and they were detected in biopsy material obtained from Rett patients as well as Rett mice (Eeg-Olofsson et al., 1990; Dotti et al., 1993; Belichenko et al., 2009). Furthermore, expression of certain subunits of respiratory complexes I and III seems to be indirectly controlled by MeCP2 (Kriaucionis et al., 2006), and just recently it has been shown that the mitochondrial overproduction of H_2_O_2_ in Rett mice is mainly due to an aberrant activity of complex II (De Filippis et al., 2015). Interestingly, oxidative stress does not only occur in MeCP2 deficiency but also in *MECP2* duplication syndrome (Signorini et al., 2016). In view of the irregular breathing and the resulting frequent intermittent hypoxic episodes in RTT, it is reasonable to assume that such transiently reduced O_2_ supply may further aggravate the conditions of already disturbed mitochondria and cellular redox imbalance.

Various oxidative stress markers—such as non-protein-bound iron, protein carbonyls, malondialdehyde (MDA) and F2-isoprostanes—are increased in blood samples of Rett patients (Sierra et al., 2001; De Felice et al., 2009, 2011, 2012b). These blood analyses also revealed a reduced activity of the pivotal scavenging enzyme superoxide dismutase (Sierra et al., 2001) as well as decreased vitamin E levels (Formichi et al., 1998). Recently, we confirmed an increased oxidative burden and mitochondrial dysfunction also in hippocampal tissue of male MeCP2-deficient (*Mecp2^−/y^*) mice (Großer et al., 2012), by taking advantage of genetically-encoded optical redox indicators which allow to quantify redox conditions and dynamics (Hanson et al., 2004; Funke et al., 2011). Interestingly, these redox- and mitochondrial alterations in MeCP2-deficient brains already start to manifest at the neonatal stage, i.e., long before erratic breathing and other characteristic Rett symptoms are evident. Therefore, increased reactive oxygen species (ROS) levels and downstream redox changes are considered pivotal elements driving the pathogenesis of RTT (Sierra et al., 2001; De Felice et al., 2009; Großer et al., 2012; Müller and Can, 2014).

The pathogenic potential arising from redox alterations may not necessarily be linked to tissue damage only. Since the activity and function of various ion channels and neurotransmitter receptors is closely controlled by ambient redox conditions (Aizenman et al., 1989; Hammarström and Gage, 2000; Müller and Bittner, 2002; Sah et al., 2002; Hidalgo et al., 2004), any transient or sustained redox imbalance may easily contribute to the disturbed function of excitatory and inhibitory synapses (Blue et al., 1999; Dani et al., 2005; Medrihan et al., 2008), the impaired synaptic plasticity (Asaka et al., 2006; Moretti et al., 2006), and the neuronal hyperexcitability (Zhang et al., 2008; Calfa et al., 2011) which are closely linked to MeCP2-deficiency. These redox-mediated changes may be transient or persisting, result from too oxidizing or too reducing conditions, and they can be expected to occur way before any oxidative tissue damage is manifesting.

In view of the mitochondrial dysfunction, the associated oxidative stress and their assumed role for the pathogenesis of RTT, improving mitochondrial function and/or reinstating cellular redox balance appear to be promising treatment concepts in RTT. Indeed, supplementation with ω-3 polyunsaturated fatty acids (PUFAs) successfully reduced the levels of several oxidative stress biomarkers in the blood of Rett patients and ameliorated some of the typical Rett symptoms (De Felice et al., 2012a; Maffei et al., 2014). Along this line, we recently evaluated *in vitro* the therapeutic merit of the free-radical scavenger Trolox, a vitamin E-derivative. In acute hippocampal slices of adult and symptomatic *Mecp2^−/y^* mice, we confirmed that acute incubation with Trolox dampens neuronal hyperexcitability, reinstates synaptic plasticity, ameliorates cellular redox balance and improves the hypoxia tolerance in *Mecp2^−/y^* hippocampus (Großer et al., 2012; Janc and Müller, 2014).

Based on these promising *in vitro* findings, we performed this preclinical trial, to define the merit of chronic Trolox treatment *in vivo*. Starting at postnatal day (PD) 10/11, Trolox was given to wild type (WT) and *Mecp2^−/y^* mice every other day by intraperitoneal (i.p.) injection for ~40 days. We screened for possible improvements in systemic and blood parameters, behavioral aspects and respiration. Markers of protein- and lipid-oxidation were quantified in isolated brain tissue, and detailed *in vitro* analyses on acute brain slices rated synaptic function and plasticity, hypoxia tolerance and mitochondrial metabolism. Also, potential changes in brain gross morphology were assessed.

## Materials and Methods

As mouse model for RTT, we used, as in our earlier studies mice lacking the *Mecp2* gene B6.129P2(C)-Mecp2^tm 1.1Bird^ (Guy et al., 2001). The entire study was performed on male mice, as *Mecp2^−/y^* mice show an earlier and more severe disease onset and present the more uniform conditions of complete MeCP2 deficiency. All procedures are in accordance with national German regulations, and were authorized by the Office of Animal Welfare of the University Medical Center Göttingen and the Lower Saxony State Office for Consumer Protection and Food Safety.

### Systemic Trolox Treatment

**Figure 1:**
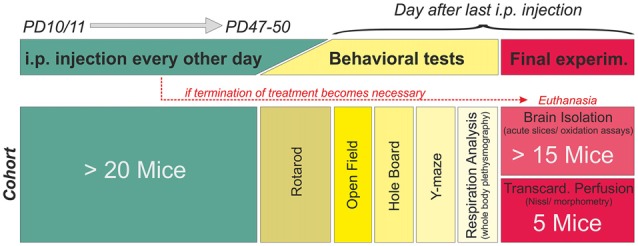
**Treatment plan and study design.** Mice were randomly assigned to a treatment (phosphate buffered saline (PBS) only, 10 mg/kg Trolox, 40 mg/kg Trolox). Injections were started PD10–11, given every 48 h, and continued up to PD47–50. Mice then underwent behavioral testing before the final experiment (tissue isolation or transcardial perfusion). The schematic refers to a single cohort, consisting of at least 20 mice. Six of these cohorts (three treatment paradigms per genotype) were run in parallel fully blinded, identified for correct treatment by color codes only. Unblinding was done after all mice had been treated and all data had been analyzed.

The systemic treatment was designed as blinded, placebo-controlled study (Figure 1). Mice were randomly assigned to a treatment group, and starting from PD10–11 they received either vehicle (phosphate buffered saline, PBS), low-dose Trolox (10 mg/kg body weight) or high-dose Trolox (40 mg/kg body weight). Compounds were administered by i.p. injection every 48 h for a duration of ~6 weeks, i.e., until the mice had reached an age at which characteristic Rett symptoms are clearly evident (~PD47–50). In case of complications (massive weight loss, infections, edema, obvious signs of pain or suffering, tremors, lethargy, ruffled fur, difficulty breathing) treatment was terminated and the respective mouse euthanized. Injections, phenotypic analyses, *in vitro* experiments and data analyses were team efforts, with some overlap among those performing injections, running experiments and analyzing data. Everyone actively involved was blinded with respect to both mouse genotype and type of treatment. Yet, in view of the severe phenotype of the *Mecp2^−/y^* mice and the differences in body weight, their genotype became more and more obvious once they reached PD30 and beyond.

Details on the pharmacokinetics of Trolox are not available, and its high blood brain barrier transferability has been demonstrated only very recently (Wada et al., 2016). Therefore, our compound dosing was chosen in accordance to earlier reports in which Trolox was i.p. injected or orally fed to rats/gerbils and successfully ameliorated neurotoxic effects as well as ischemia-related neurodegeneration (Gupta and Sharma, 2006; Al Mutairy et al., 2010). At the same time, the higher Trolox dose of 40 mg/kg is the consequence of the maximum soluble amount in saline (~4 mg/ml without additional solvents) and the maximum allowable volume for i.p. injections in mice. The 48 h injection interval is based on a report on mice, which shows that upon administration, the levels of the vitamin E compound tocopherol in blood, brain, lung, kidney and liver remain stable for at least 24 h upon a single dose administration (Baxter et al., 2012). Furthermore, we were concerned that i.p. injections performed on a daily basis for several weeks—especially in view of the early onset of treatment (PD10/11)—would be too stressful for the fragile Rett mice.

### Solutions

All chemicals and assays were obtained from Sigma-Aldrich, unless indicated otherwise. Trolox ((±)-6-hydroxy-2,5,7,8-tetramethylchromane-2-carboxylic acid) was dissolved in PBS (1 mg/ml and 4 mg/ml injection solution), stored at 4°C, and used within 1 week. Artificial cerebrospinal fluid (ACSF) required for *in vitro* experiments contained (in mM): 130 NaCl, 3.5 KCl, 1.25 NaH_2_PO_4_, 24 NaHCO_3_, 1.2 CaCl_2_, 1.2 MgSO_4_ and 10 glucose. Constant aeration with carbogen (95% O_2_, 5% CO_2_) adjusted pH to 7.4. Cyanide (CN^−^) was dissolved as aqueous 1 M stock and stored at −20°C. FCCP (carbonyl cyanide-4-(trifluoromethoxy) phenylhydrazone, Tocris Bioscience) and rhodamine 123 (Rh123) were dissolved as 10 mM stocks in dimethyl sulfoxide (DMSO) and stored at 4°C. Final DMSO concentration was ≤0.02%.

### Behavioral Testing and Whole-Body Plethysmography

For detailed phenotyping, all mice underwent various behavioral tests prior to final experiments, to rate motor function and activity, environmental exploration, and working memory. However, to prevent an “overtesting” not every animal underwent each test available, but typically only three of the behavioral tests. Motor coordination was tested on three consecutive days (~PD43), using a mouse Rotarod (Ugo Basile). The first day allowed the mice to adapt to the treadmill, the second day defined motor performance, and improvement from the second to the third day represents motor learning. Mice were placed on the rotating drum (non-skid surface, 30 mm diameter), the speed continuously accelerated from 5 rpm to 50 rpm within 5 min, and the time each animal stayed on the rod was measured as latency to fall. Occasionally, mice clasped to the rotating drum without running. In these cases the timing was stopped after five full rotations of clasping.

Motor activity and environmental exploration were rated in open field and hole board tests (~PD47–50). In the open field (45 cm × 45 cm), the motility of mice was monitored for a duration of 5 min. Any lateral movements were detected by a grid of 16 infrared beams and tracked using ActiTrack v2.7.13 software (Panlab, Harvard Apparatus). For detailed analyses, the open field was divided in three zones (a 95 mm wide periphery, the respective 95 mm × 95 mm corners and the remaining 260 mm × 260 mm center) which were defined as a virtual mask in the tracking software (see Figure 3B). In the hole board configuration, a base-plate with 16 equally spaced holes (25 mm diameter) was inserted into the arena, and an additional grid of infrared beams detected the head-dipping behavior of the mice.

Working memory was analyzed in a Y-maze (32.5 cm long and 8.5 cm wide arms, equally spaced at 120° angles), in which mice were tracked by video camera and ANY-maze software (Stoelting). Around PD47–50, mice were placed in the start arm and allowed to explore the maze for 10 min while one arm was blocked. After 1 h the test was repeated for a duration of 5 min with all arms being accessible, and the number of spontaneous alterations was counted. An alternation is defined as consecutive entry of all three arms in any order. Thus, the number of maximum possible alternations is the total sum of arm entries minus two. To compare mice, the number of spontaneous alternations was normalized to the maximum possible alternations.

To analyze breathing (PD47–50), mice were placed in a whole-body plethysmograph (Data Sciences International) and allowed to breathe naturally under conscious and unrestrained conditions. Breathing patterns were recorded by Ponemah v5 control software. After a ~10 min adaptation, breathing was analyzed for the following 3 min. The individual respiratory cycles were detected by the threshold crossing method (Clampfit 10.3, Molecular Devices), and breathing frequencies calculated as the reciprocal of the averaged inspiratory interval. The irregularity score was determined as the normalized difference between a pair of subsequent breaths (Barthe and Clarac, 1997; Telgkamp et al., 2002; Wegener et al., 2014).

### Preparation and Transcardial Perfusion

To obtain brain tissue, mice (~PD50) were deeply anesthetized with ether and decapitated. The brain was isolated and placed in chilled ACSF for ~2 min. Coronal brain slices of 400 μm thickness were cut using a vibroslicer (752M Vibroslice, Campden Instruments) and split along the sagittal midline. Depending on the experiment they were transferred to an interface recording chamber or a submersion-style storage chamber. Prior to the experiments, slices were left undisturbed for at least 90 min, to ensure their recovery from dissection and slicing. Any brain tissue not required for acute analyses was immediately frozen in liquid nitrogen for later biochemical assays. Blood samples collected upon decapitation served to determine hematocrit and blood glucose levels (Contour^®^ blood glucose meter; Bayer).

For transcardial perfusion, deep ether anesthesia was confirmed by the absence of foot-pinch reflexes. The chest was opened, the heart exposed, and an injection needle inserted in the left ventricle. Remaining blood was removed from the vascular system by PBS perfusion, then perfusion fixation was performed by 4% paraformaldehyde (PFA) in PBS. Once isolated, brains were postfixed for 3 days in 4% PFA and long-term stored in PBS with 0.02% NaN_3_.

### Nissl Staining

Perfusion-fixed brains were cut into coronal sections (30 μm) using a microtome (VT1200S Leica). The sections were placed on microscope slides, passed through a descending ethanol series (95% for 15 min; 70% and 50% for 1 min each) and washed twice in demineralized water. They were then stained in an aqueous cresyl violet/acetate solution (5 mg/ml cresyl violet, 30 μl/ml acetic acid) for 2 min. Remaining dye was washed out by water, followed by an ascending ethanol series (50% for 1 min, 70% plus 1% acetic acid for 2 min, 95% for 2 min, 99.9% for 2 min). Finally, sections were cleared in xylol (5 min), dried (15 min), and coverslipped with Eukitt quick-hardening mounting medium. For the morphometric studies a rostrocaudal location of about −1.7 mm with respect to Bregma was chosen, and three sections per brain were analyzed using a digital microscope (Coolscope, Nikon) with NIS-Elements 3.2 cell analysis software. Quantification of neuronal size is based on soma area, and it was determined by randomly selecting five cells in each section analyzed. Those cells were chosen, which showed clearly identifiable boundaries and were not obstructed by neighboring/overlapping objects. The cross-sectional area was determined in the best focal plane by drawing along the outer cell boundaries (NIS-Elements analysis software).

### Electrophysiological Recordings

Electrophysiological recordings were run in an Oslo style interface chamber, which was constantly aerated with carbogen (95% O_2_, 5% CO_2_; 400 ml/min) and supplied with oxygenated ACSF (3–4 ml/min). Synaptic plasticity and hypoxic responses were analyzed at temperatures of 31–32°C and 35–36°C, respectively. Recording electrodes were pulled from borosilicate capillaries (GC150TF-10, Harvard Apparatus) on a horizontal electrode puller (Model P-97, Sutter Instruments) and filled with ACSF. Their tip resistance was trimmed to ~5 MΩ. Schaffer collaterals were stimulated with stainless steel electrodes (50 μm diameter microwire, AM-Systems), delivering 0.1 ms unipolar stimuli (S88 stimulator with PSIU6 stimulus isolation units, Grass Instruments). Field excitatory postsynaptic potentials (fEPSPs) and extracellular DC potentials were recorded in CA1 *st. radiatum*, using a locally constructed field potential amplifier (Hepp et al., 2005). Data were sampled with an Axon Instruments Digitizer 1322A and analyzed by PClamp 9.2 software (both Molecular Devices). Synaptic function was rated based on input/output (i/o) curves and long-term potentiation (LTP). For LTP experiments, stimulus intensity was set to obtain half-maximum responses. Stable LTP was induced by three subsequent trains of high-frequency stimulation (100 Hz, 1 s, 5 min train interval; Janc and Müller, 2014). Hypoxia-induced spreading depression (HSD) was elicited by switching the interface chamber’s gas supply to nitrogen (95% N_2_ and 5% CO_2_); O_2_ was resubmitted 30 s after HSD onset (Hepp et al., 2005).

### Optical Recordings

Mitochondrial metabolism and membrane potentials were monitored in CA1 *st. radiatum* using computer-controlled epifluorescence imaging systems. They were composed of a polychromatic xenon-light source (Polychrome II, Till Photonics), a CCD-camera (SensiCam QE, PCO) and an upright microscope (either Axiotech or Axioskop I, Zeiss). Hippocampal slices were imaged in a submersion-style chamber (30–33°C), using a 40× 0.8NA water immersion objective (Achroplan Zeiss). FAD and NADH tissue autofluorescence were monitored in a ratiometric approach (Duchen and Biscoe, 1992), by alternating excitation at 360 nm (NADH, 70 ms exposure) and 445 nm (FAD, 40 ms exposure). Image pairs were taken every 5 s (0.2 Hz frame rate), and autofluorescence was separated by a 450 nm dichroic mirror and a 510/80 nm emission bandpass filter (Gerich et al., 2006; Janc and Müller, 2014).

For Rh123 imaging of mitochondrial membrane potential changes, slices were dye-loaded (5 μM, 15 min) in a miniaturized staining chamber (Funke et al., 2007). As Rh123 was used in quenching mode, depolarization of mitochondria is indicated by an increase in Rh123 fluorescence (Emaus et al., 1986; Foster et al., 2006). Rh123 was excited at 480 nm using frame rates of 0.2 Hz and 5 ms exposure times; its emission was separated using a 505 nm dichroic mirror and a 535/35 nm bandpass emission filter.

### Lipid Peroxidation and Protein Carbonylation

To assess oxidative tissue damage, lipid peroxidation and protein carbonylation were quantified in cryopreserved tissue samples of frontal cortex. Lipid peroxidation was determined in ~10 mg neocortical tissue using a colorimetric assay (MAK085), which is based on the reaction of MDA with thiobarbituric acid (TBA). Tissue samples were homogenized on ice in 300 μl MDA lysis buffer containing butylhydroxytoluene (100×), and debris was removed by centrifugation (13,000 g, 10 min). Then 0.2 ml of homogenate was added to 600 μl TBA solution. This mixture was incubated for 60 min at 95°C, cooled down on ice to room temperature (RT), and centrifuged again (13,000 g, 30 s) before absorbance at 532 nm was determined against a MDA standard curve.

Protein oxidation was determined using a protein carbonyl assay (MAK094), which reports the interaction of protein carbonyl groups with 2,4-dinitrophenylhydrazine (DNPH). The resulting stable dinitrophenyl (DNP) hydrazone adducts are detected spectrophotometrically. Tissue samples were homogenized in 500 μl CelLytic M buffer and incubated for 15 min before debris was removed by centrifugation (14,000 g, 15 min). The supernatant was collected and its protein content determined by Bradford assay (B6916). To quantify protein carbonyl levels, 0.15 ml of tissue supernatant (~10 mg/ml protein) was mixed with 0.15 ml of DNPH solution and incubated at RT for 10 min. Then, protein was precipitated by adding 0.45 ml of 100% trichloroacetic acid (5 min incubation on ice) and subsequent centrifugation at 13,000 g for 2 min. The pellet was dissolved in ice-cold acetate, incubated for 5 min at −20°C and centrifuged again at 13,000 g for 2 min, before the previous acetate washing step was repeated. Then the precipitated protein was re-dissolved in 0.33 ml of 6 M guanidine solution and incubated for 20 min at 60°C. Samples were allowed to cool down to RT; their carbonyl content was calculated from the absorbance at 375 nm, and their final total protein content determined by bicinchoninic acid assay (BCA1 AND B9643).

### Statistics

For electrophysiological and optical recordings slices of at least five different brains of each genotype and treatment group were used. In behavioral experiments at least six mice per group were tested. All numerical values are given as mean ± standard deviation; the number of experiments (n) refers to the number of slices or mice analyzed. The significance of the changes observed was tested by two-way ANOVA followed by *post hoc* all-pairwise multiple comparisons (Holm-Sidak method); statistical calculations were performed with Sigmaplot 12.5 (Systat Software Inc.). In the diagrams, statistically significant differences among treatment groups of the same genotype are indicated by asterisks (^*^P < 0.05, ^*^^*^P < 0.01; ^*^^*^^*^P < 0.001), and those among WT and *Mecp2^−/y^* mice are identified by crosshatches (^#^*P* < 0.05, ^##^*P* < 0.01, ^###^*P* < 0.001).

## Results

To define the pharmacological merit of a systemic free-radical scavenger treatment in a mouse model of RTT, we assessed various systemic and behavioral parameters and conducted an array of *in vitro* analyses on the brain tissue of the treated mice.

### Systemic Parameters

*In vivo* treatment started at the presymptomatic stage (PD10–11), to detect potential differences in disease onset or progression. Injections of either PBS, low-dose (10 mg/kg) or high-dose Trolox (40 mg/kg) were performed every 48 h for ~6 weeks (Figure 1). In total, 177 mice (88 WT and 89 *Mecp2^−/y^* mice*)* received blinded Trolox or PBS injections for the entire treatment duration. Another 17 mice (3 WT, 14 *Mecp2^−/y^*) died or had to be euthanized before the end of treatment. The deceased WT mice had received 10 mg/kg (*n* = 2) and 40 mg/kg Trolox (*n* = 1); the deceased *Mecp2^−/y^* mice belonged to PBS (*n* = 1), low-dose Trolox (*n* = 7) and high-dose Trolox-treated groups (*n* = 6). For summary and detailed survival rates see Table 1.

**Table 1:** **Detailed overview of the six treatment groups, their group sizes and the relative survival rates reported as % values of total group size**.

	Treatment	Survived	Deceased	Total
WT	PBS only	29 (100%)	0 (0%)	29
	10 mg/kg Trolox	24 (92.3%)	2 (7.7%)	26
	40 mg/kg Trolox	35 (97.2%)	1 (2.8%)	36
*Mecp2^−/y^*	PBS only	30 (96.8%)	1 (3.2%)	31
	10 mg/kg Trolox	33 (82.5%)	7 (17.5%)	40
	40 mg/kg Trolox	26 (81.2%)	6 (18.8%)	32

**Figure 2:**
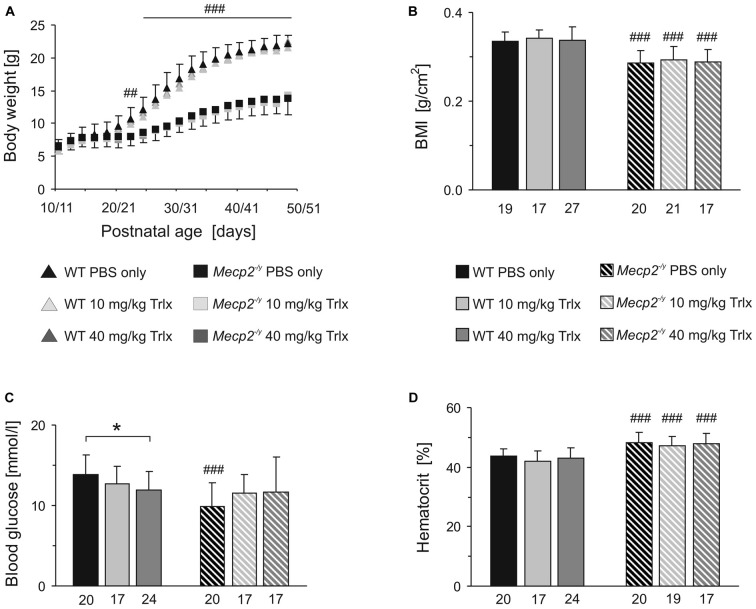
**Trolox treatment does not improve growth and hematocrit of *Mecp2^−/y^* mice, but abolishes the genotypic differences in blood glucose levels. (A)** Body weights of *Mecp2^−/y^* and wild type (WT) mice were indistinguishable at first. Yet, from the third postnatal week, all *Mecp2^−/y^* groups gained less weight. Plotted are averaged body weights of 24–35 mice. For clarity, standard deviations are included for the PBS-treated groups only. Crosshatches indicate significant differences between *Mecp2^−/y^* and the respective WT mice (^##^*P* < 0.01; ^###^*P* < 0.001). **(B)** The body mass index (BMI) determined at PD47–50 was significantly lower in *Mecp2^−/y^* than in WT mice. Bar shading and patterns also apply to panels **(C,D)**. The respective number of animals analyzed is indicated below each bar. **(C)** *Mecp2^−/y^* mice receiving PBS showed a lower blood glucose level than PBS-treated WTs. Low- and high-dose Trolox treatment tended to increase glucose levels in *Mecp2^−/y^* mice and abolished the genotypic differences. In WTs, high-dose Trolox significantly reduced the blood glucose content. This genotype-matched difference is indicated by asterisks (^*^P < 0.05). **(D)** The increased hematocrit of *Mecp2^−/y^* mice was not affected by any treatment.

In terms of body weights, WT and *Mecp2^−/y^* mice were indistinguishable up to the third postnatal week. Yet, from PD22 on, all *Mecp2^−/y^* mice—independent of the treatment—showed a reduced growth and gained less body weight than the WTs (Figure 2A). Their final body weight at PD50 was about half the weight of WT mice, and their body mass index (BMI, determined as body weight divided by body length^2^) was also significantly lower (Figure 2B). Genotype-matched comparison did not reveal any effects of the type of treatment on the growth parameters among the different WT or *Mecp2^−/y^* groups.

Blood analyses detected ~29% lower blood glucose levels in PBS-treated *Mecp2^−/y^* mice than in the corresponding WTs. Those *Mecp2^−/y^* mice receiving low or high Trolox doses, showed a trend towards higher glucose levels and they did no longer differ significantly from PBS-treated or Trolox-receiving WTs (Figure 2C). In contrast, Trolox tended to reduce glucose levels in WTs, which reached the level of significance in the high-dose treated group (Figure 2C). The increased hematocrit, which we reported already earlier for *Mecp2^−/y^* mice (Fischer et al., 2009), was still evident in all *Mecp2^−/y^* groups; any modulation by Trolox was not observed (Figure 2D).

### Behavioral Testing

**Figure 3:**
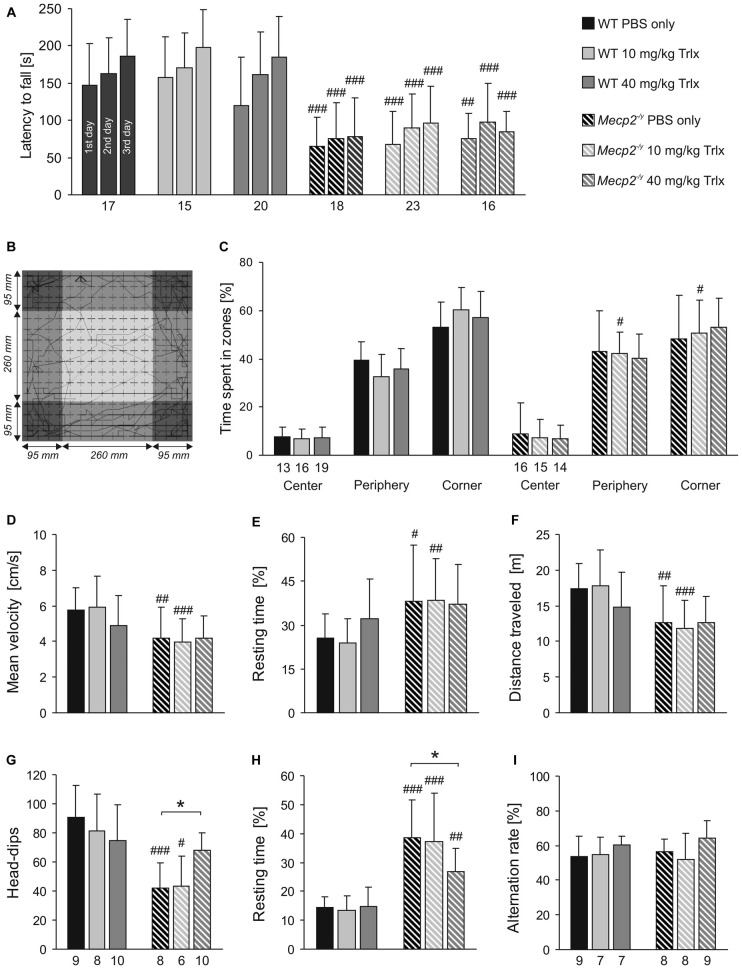
**Trolox-treated *Mecp2^−/y^* mice still show limited motor skills and reduced activity. (A)** In Rotarod tests, the latency to fall was determined on three consecutive days. All *Mecp2^−/y^* mice showed a weaker motor performance than WTs, and Trolox did not affect motor performance in either genotype. **(B)** Open field tests rated general locomotor activity and anxiety. The arena was divided in the three virtual zones center, periphery and corner. The sample track represents a 5 min run, and the dimensions of the arena and of the three virtual zones are indicated. **(C)** The relative times spent in the three zones did not markedly differ among *Mecp2^−/y^* and WT groups, nor was it considerably affected by the type of treatment. **(D–F)** *Mecp2^−/y^* mice treated with PBS or low-dose Trolox traveled at lower velocities, rested longer and ran shorter distances than the respective WTs. In high-dose Trolox injected mice, these genotypic differences were not detectable anymore, as the treatment tended to dampen the activity of WT mice. **(G)** Hole-board testing revealed fewer head-dips in *Mecp2^−/y^* mice treated with PBS or low-dose Trolox. In contrast, *Mecp2^−/y^* mice receiving high-dose Trolox showed more frequent head-dips than the other *Mecp2^−/y^* groups, and they did no longer differ from the corresponding WTs. **(H)** The resting time was shortened in *Mecp2^−/y^* mice receiving high-dose Trolox. Nevertheless, all *Mecp2^−/y^* groups rested significantly longer than the respective WTs. **(I)** Y-maze testing did not detect any differences between WT and *Mecp2*^−/y^ mice. Asterisks identify genotype-matched differences (^*^P < 0.05), crosshatches indicate differences between the respective *Mecp2^−/y^* and WT groups (^#^*P* < 0.05, ^##^*P* < 0.01, ^###^*P* < 0.001).

Rett patients and Rett mice show complex motor impairment. To assess whether Trolox may exert any positive effects on motor coordination, rotarod tests were performed on three consecutive days (Figure 3A). After habituation (first day), all *Mecp2^−/y^* groups showed a poorer performance on the rotarod on the second day and only stayed on the treadmill for markedly ( >40%) shorter intervals than the respective WTs. Motor learning is expected between the second and third day of testing. Indeed, WT mice regardless of the treatment showed a trend towards increased running times of ~20%. In the *Mecp2^−/y^* groups, any improvement of motor learning between the second and third day was not seen (Figure 3A).

Exploratory behavior and locomotor activity of mice was determined in an open field arena (Figure 3B). This test mimics the natural conflict of mice between exploring a novel environment and avoiding illuminated, open areas. All mice avoided the center region, rather preferring the periphery and even more the corners (Figures 3B,C). Marked differences in the relative times spent within the three zones were not obvious between *Mecp2^−/y^* and WT mice. *Mecp2^−/y^* mice treated with PBS and low-dose Trolox were significantly less active than WTs, as indicated by their reduced average velocity (Figure 3D), longer relative resting time (Figure 3E), and shorter total distance traveled (Figure 3F). Interestingly, these activity parameters did not significantly differ among high-dose Trolox-treated WT and *Mecp2^−/y^* mice, as WTs tended to become less active under this treatment (Figures 3D,E,F).

Environmental exploration of mice was tested by the hole board. *Mecp2^−/y^* mice receiving PBS or low-dose Trolox showed significantly fewer head-dips than the respective WTs and rested longer (Figures 3G,H). High-dose Trolox-treated *Mecp2^−/y^* mice became clearly more active than the other *Mecp2^−/y^* groups, showing more head-dips and resting less than PBS-treated *Mecp2^−/y^* mice. Also their head dipping did no longer differ from high-dose treated WTs (Figure 3G).

For the assessment of spatial working memory, the Y-maze is considered a useful initial test (Dudchenko, 2004). It can be performed relatively easily, does not require multiple repeats, and does not impose high levels of stress or pronounced motor challenge on the mice. In view of the clear learning/memory deficits of MeCP2-deficient mice (Pelka et al., 2006) and their motor impairment, we therefore established this test. Against expectation, the number of spontaneous alterations did not differ among the respective WT and *Mecp2^−/y^* groups, nor did genotype-matched comparison indicate any effects of Trolox (Figure 3I). This suggests that the spatial complexity of only three arms is not sufficiently challenging to detect clear genotypic effects.

**Figure 4:**
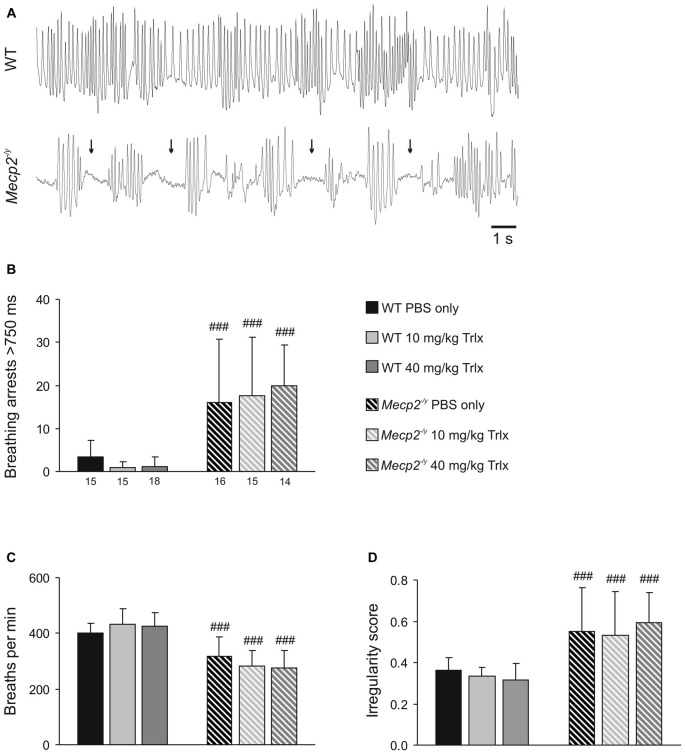
**Irregular breathing of *Mecp*2^−/*y*^ mice persists upon Trolox treatment. (A)** Breathing pattern typically seen during whole-body plethysmography in WT and *Mecp2^−/y^* mice. The recordings were obtained from PBS-treated mice; note the frequent breathing arrests in the *Mecp2^−/y^* mouse (arrows). **(B)** In all *Mecp2^−/y^* groups, breathing arrests were far more frequent than in WTs. Plotted is the total number of breathing arrests exceeding 750 ms within a time period of 3 min. **(C)** Averaged breathing frequencies were significantly lower in all *Mecp2^−/y^* mice than in WTs. **(D)** Severe breathing disturbances in *Mecp2^−/y^* mice are evident as a noticeably increased irregularity score, which persisted despite Trolox treatment. Differences between the respective *Mecp2^−/y^* and WT groups are indicated by crosshatches (^###^*P* < 0.001).

To evaluate the breathing pattern, unrestrained whole-body plethysmography was performed. Breathing was clearly disturbed in PBS-treated *Mecp2^−/y^* mice as compared to WTs (Figure 4A), and Trolox treatment failed to improve the regularity of breathing in *Mecp2^−/y^* mice. All *Mecp2^−/y^* groups still showed a higher incidence of breathing arrests and lower breathing frequencies than WTs (Figures 4B,C). Accordingly, the irregularity score, i.e., the variability of respiratory cycle duration, remained increased in all *Mecp2^−/y^* mice (Figure 4D).

### Hypoxia Tolerance

**Figure 5:**
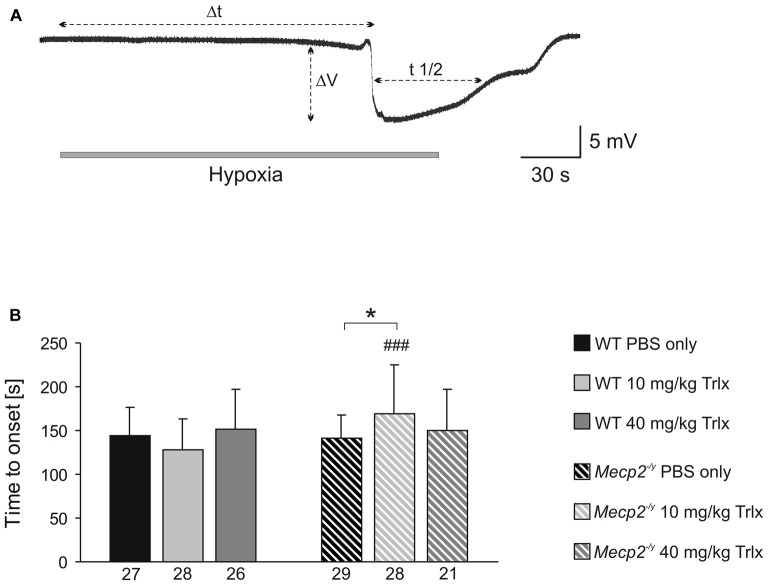
**Low-dose Trolox increases the hypoxia tolerance of *Mecp*2^−/*y*^ hippocampus. (A)** Negative extracellular DC potential shift associated with the occurrence of hypoxia-induced spreading depression (HSD). Recording was performed in CA1 *st. radiatum* of an acute slice of a PBS-treated *Mecp2^−/y^* mouse. The characteristic parameters of the DC potential shift (Δt, time to onset; ΔV, amplitude; t1/2, duration) are defined. **(B)** Low-dose Trolox treatment postponed HSD onset in *Mecp2^−/y^* slices as compared to the respective WTs and PBS-treated *Mecp2^−/y^* mice. Asterisks identify genotype-matched differences (^*^P < 0.05), crosshatches indicate differences between the respective *Mecp2^−/y^* and WT groups (^###^*P* < 0.001).

To screen for differences in hypoxia susceptibility, acute hippocampal tissue slices isolated from treated WT and *Mecp2^−/y^* mice were exposed to transient O_2_ withdrawal to trigger an HSD episode, which represents the concerted network response to severe hypoxia. HSD was recorded as negative extracellular DC-potential shift, and its characteristic parameters amplitude (ΔV), duration at the half amplitude level (*t*_1/2_), and time to onset (Δt) were analyzed (Figure 5A). In hippocampal slices of low-dose Trolox-treated *Mecp2^−/y^* mice, the time to HSD onset was significantly increased as compared to the respective WTs and PBS-treated *Mecp2^−/y^* mice (Figure 5B). The amplitude and duration of the DC potential shift did not differ among genotypes and treatment groups (Table 2).

**Table 2:** **Summary of the characteristic hypoxia-induced spreading depression (HSD) parameters for the different wild type (WT) and *Mecp2*^−/*y*^ groups**.

	Treatment	WT	*Mecp2 ^−/y^*
Time to onset [s]	PBS only	144.2 ± 32.9 (*n* = 27)	140.7 ± 26.8 (*n* = 29)
	10 mg/kg Trolox	127.8 ± 35.3 (*n* = 28)	168.7 ± 57.0 (*n* = 28)
	40 mg/kg Trolox	151.5 ± 45.8 (*n* = 26)	150.4 ± 46.4 (*n* = 21)
	Naïve slices	160.0 ± 39.3 (*n* = 4)	116.0 ± 19.3 (*n* = 4)
Amplitude [mV]	PBS only	−14.6 ± 2.8	−15.7 ± 3.5
	10 mg/kg Trolox	−14.7 ± 3.9	−14.4 ± 3.3
	40 mg/kg Trolox	−14.5 ± 2.5	−14.1 ± 4.0
	naïve slices	−15.9 ± 3.3	−18.4 ± 2.7
Duration [s]	PBS only	69.5 ± 12.7	65.0 ± 13.6
	10 mg/kg Trolox	63.4 ± 13.1	62.6 ± 10.3
	40 mg/kg Trolox	70.6 ± 20.0	67.4 ± 11.1
	Naïve slices	74.1 ± 9.7	63.1 ± 3.5

*The number of slices analyzed is reported. The group of naïve slices represents control tissue isolated from otherwise untreated and non-handled mice*.

Against expectation, the earlier observed increased hypoxia susceptibility of *Mecp2^−/y^* hippocampal slices (Fischer et al., 2009; Janc and Müller, 2014) was not obvious among PBS-treated WT and *Mecp2^−/y^* mice. To clarify, whether the lack of this genotypic difference may be due to frequent animal handling and i.p. injections, seasonal or breeding issues, HSD was triggered also in slices from untreated (naïve) *Mecp2^−/y^* and WT mice. These controls reproduced the hastened onset of HSD by ~28% in slices from *Mecp2^−/y^* mice with almost the identical times to HSD onset (Table 2) reported previously for naïve *Mecp2^−/y^* and WT slices (Janc and Müller, 2014; Table 3).

**Table 3:** **Genotypic differences among WT and *Mecp2^−/y^* mice that either underwent PBS (placebo only) treatment or were not injected and handled frequently (naïve mice)**.

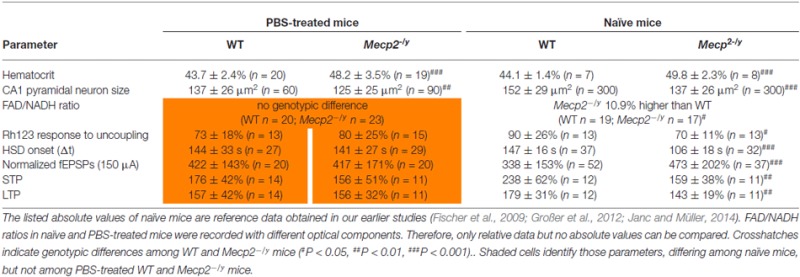

### Synaptic Function and Plasticity

**Figure 6:**
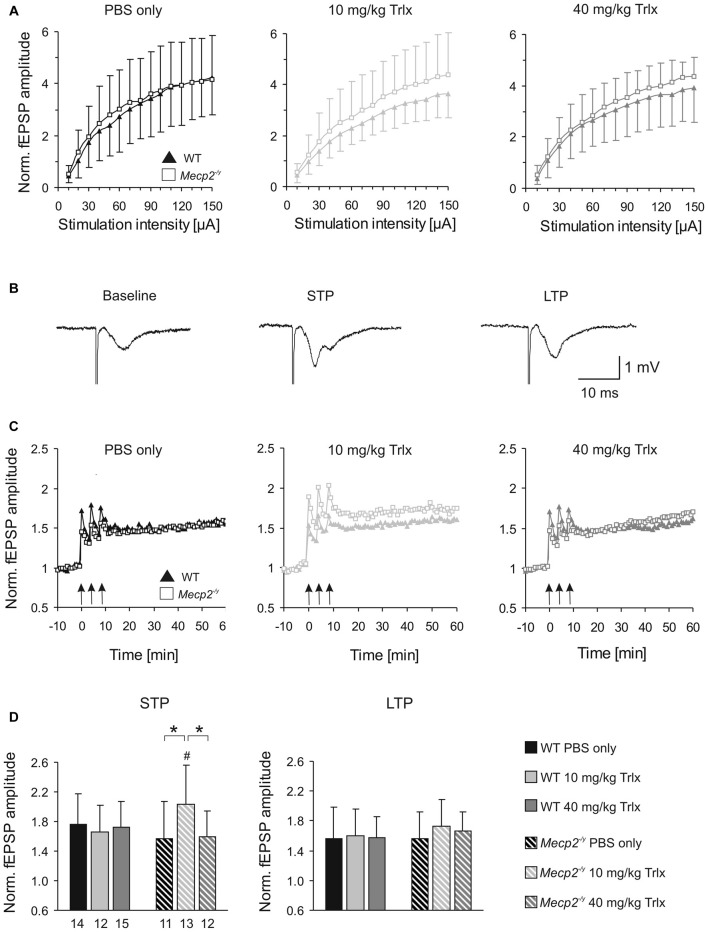
**Improved synaptic plasticity in *Mecp2*^−/y^ mice after low-dose Trolox treatment. (A)** Input/output (i/o) curves of all treatment groups recorded in acute hippocampal slices. Plotted field excitatory postsynaptic potential (fEPSP) amplitudes are normalized to the fiber volley, representing the averages of 20–27 slices. Significant genotypic differences or treatment effects on neuronal excitability were not found, but Trolox treated WT mice tended to show somewhat reduced fEPSP amplitudes. **(B)** Sample fEPSPs of PBS-treated WT mice under baseline conditions, immediately after high-frequency stimulation (short-term potentiation, STP), and 1 h after inducing long-term potentiation (LTP). Stimulation artifacts are truncated. **(C)** STP was improved and the degree of LTP tended to increase in low-dose Trolox-treated *Mecp2^−/y^* mice as compared to the respective WT group. For clarity, error bars are omitted. Synaptic potentiation (STP, LTP) was induced by three consecutive 100 Hz stimulation trains (arrow marks), lasting 1 s each. **(D)** Comparing STP and LTP for the different groups shows that STP is significantly improved only in low-dose Trolox-treated *Mecp2^−/y^* mice. Further differences among genotypes and/or treatment groups were not found. Asterisks identify genotype-matched differences (^*^P < 0.05), crosshatches indicate differences between the respective *Mecp2^−/y^* and WT groups (^#^*P* < 0.05).

Synaptic function and plasticity were assessed in acute hippocampal tissue slices by recording fEPSPs. Basal synaptic function, as judged on normalized i/o curves, did not noticeably differ between PBS-treated WT and *Mecp2^−/y^* mice (Figure 6A). The pronounced hyperexcitability observed earlier in slices of naïve *Mecp2^−/y^* mice (Janc and Müller, 2014), was not present among the PBS-injected groups. Neither did Trolox treatment induce any significant effects on basal synaptic function (Figure 6A). Trolox treated WT mice showed, however, a trend towards reduced fEPSP amplitudes.

Synaptic plasticity was rated upon high-frequency stimulation, which mediated a robust short-term potentiation (STP) and LTP of fEPSPs in all *Mecp2^−/y^* and WT groups, regardless of treatment (Figures 6B,C). The amount of STP, determined right after the third stimulus train, was significantly more pronounced in low-dose Trolox-treated *Mecp2^−/y^* mice than in the respective WTs and in *Mecp2^−/y^* mice receiving PBS or high Trolox (Figure 6D). Other differences between genotypes and/or treatment groups were not detected. The degree of LTP (determined at 50–60 min upon train stimulation) did not differ significantly among the various groups, but the final level of LTP reached, tended to be higher in low-dose Trolox-treated than in PBS injected *Mecp2^−/y^* mice (Figure 6D).

### Mitochondrial Metabolism and Function

**Figure 7:**
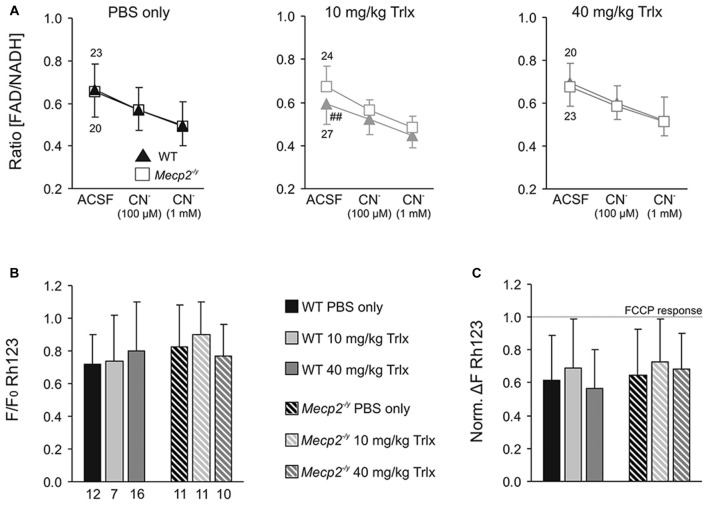
**Systemic Trolox treatment does not in general interfere with mitochondrial function. (A)** Recording the ratio of FAD/NADH autofluorescence in hippocampal slices did not identify consistent differences in basal mitochondrial respiration (artificial cerebrospinal fluid, ACSF) and in the responses to CN^−^-induced anoxia among *Mecp2^−/y^* and WT mice or the different treatment groups. Note however, that low-dose treated WTs showed lower FAD/NADH baseline levels than the corresponding *Mecp2^−/y^* group (^##^*P* < 0.01). Error bars are plotted in one direction only (WT down, *Mecp2^−/y^* up), and the number of slices analyzed is indicated. **(B)** The mitochondrial membrane potential responses to FCCP-induced uncoupling, measured as an increase in rhodamine 123 (Rh123) fluorescence, did not differ among the various groups. Therefore, major differences in mitochondrial polarization can be ruled out. **(C)** Chemical anoxia, induced by 100 μM CN^−^, elicited a pronounced mitochondrial depolarization, plotted here in reference (normalized) to the maximum FCCP-induced depolarization. These anoxia-mediated increases in Rh123 fluorescence did not differ among genotypes or treatment groups, suggesting similar mitochondrial vulnerabilities to mitochondrial challenge.

RTT is accompanied by mitochondrial alterations. To assess whether Trolox-treatment may mediate any effects on mitochondrial metabolism, we monitored FAD/NADH autofluorescence as well as Rh123 fluorescence in acute hippocampal tissue slices of the treated mice. In correspondence to the electrophysiological recordings, these imaging experiments were performed in CA1 *st. radiatum*. The basal ratio of FAD/NADH autofluorescence did not differ among PBS and high-dose Trolox treated *Mecp2^−/y^* and WT mice; but it was slightly decreased in low-dose Trolox-treated WTs (Figure 7A). Pharmacological inhibition of mitochondrial respiration by low (100 μM) and high (1 mM) doses of CN^−^ provoked the expected decreases in FAD/NADH ratio, whose magnitudes were similar among treatment groups and genotypes (Figure 7A).

Mitochondrial polarization and anoxia vulnerability were assessed in Rh123-loaded slices, by evoking maximum depolarization with the uncoupler FCCP (5 μM, 5 min) and blocking mitochondrial respiration with CN^−^ (100 μM, 2 min), respectively. The maximum mitochondrial depolarization, indicated by a massive increase in Rh123 fluorescence, did not differ among genotypes and treatment groups (Figure 7B). Neither did CN^−^-mediated inhibition of respiration evoke any different mitochondrial depolarizations (Figure 7C). It therefore can be concluded that Trolox treatment—even when applied systemically over a longer period—does not generally interfere with mitochondrial metabolism and function.

### Lipid Peroxidation and Protein Carbonylation

The redox alterations associated with RTT were reported to culminate in oxidative damage in the brains of Rett mice (De Felice et al., 2014). Therefore, we determined the extent of lipid peroxidation and protein carbonylation in brain tissue of the treated mice (~PD50), to rate the antioxidative capacity of Trolox. These tests were performed on neocortex, as the hippocampi had already been used for the previously described FAD/NADH and Rh123 imaging experiments. First, we verified the feasibility of the chosen assays, and incubated neocortical tissue samples with the oxidant tert-butyl hydroperoxide (TBHP; 500 μM, 10 min) to induce oxidative damage. As compared to control tissue, TBHP increased lipid peroxidation and protein carbonyl content by 59.4 ± 8.2% and 79.7 ± 16.1% (each *n* = 3), respectively, confirming that both assays are functional.

**Figure 8:**
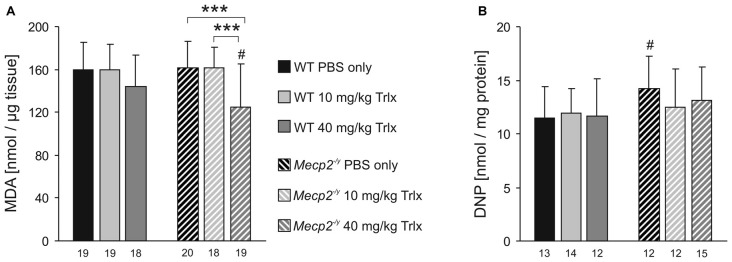
**Trolox dampens oxidative tissue damage in *Mecp*2^−/*y*^ neocortex. (A)** The malondialdehyde (MDA) level, a marker for lipid peroxidation, did not differ among PBS- or low-dose Trolox-treated WT and *Mecp2^−/y^* mice. Yet, *Mecp2^−/y^* mice receiving high-dose Trolox, showed significantly lower MDA levels than the PBS- and low-dose Trolox treated *Mecp2^−/y^* group and high-dose Trolox treated WTs. **(B)** Protein carbonylation, an indicator for oxidative damage of proteins, was higher in PBS-treated *Mecp2^−/y^* mice than in WTs. Among the Trolox-treated groups, this genotypic difference was no longer present. Asterisks identify genotype-matched differences (^***^P < 0.001), crosshatches indicate differences between the respective *Mecp2^−/y^* and WT groups (^#^*P* < 0.05).

Comparing neocortical samples from PBS-treated WT and *Mecp2^−/y^* mice, did not reveal any genotypic differences in lipid peroxidation (Figure 8A). In *Mecp2^−/y^* mice treated with high-dose Trolox, however, the degree of lipid peroxidation was significantly dampened as compared to PBS- and low-dose Trolox injected *Mecp2^−/y^* mice and high-dose Trolox treated WT mice (Figure 8A). In WTs, high-dose Trolox just showed a tendency to decrease lipid peroxidation. Protein carbonyl content was increased in PBS-treated *Mecp2^−/y^* mice as compared to the corresponding WTs, whereas in low- and high-dose Trolox treated *Mecp2^−/y^* mice this genotypic difference was no longer detectable (Figure 8B). Accordingly, the degree of oxidative damage in brain tissue can be ameliorated at least partly by Trolox treatment.

### Morphological Analyses

**Figure 9:**
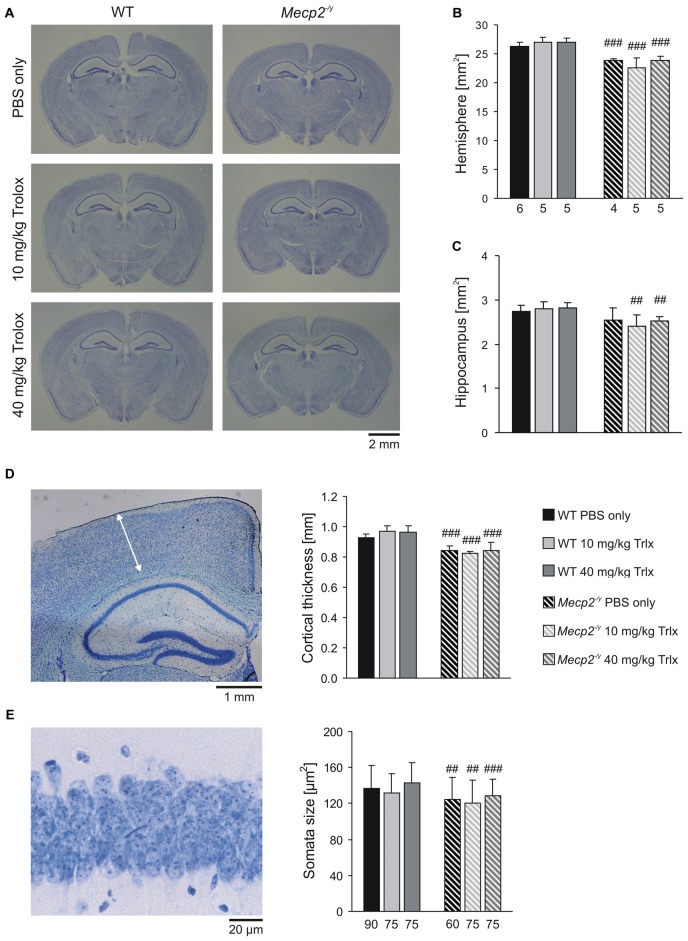
**Microcephaly in *Mecp2^−/y^* mice is not ameliorated by Trolox treatment. (A)** Nissl staining of sections (30 μm) from perfusion-fixed brains revealed the expected differences in brain size among WT and *Mecp2^−/y^* mice. **(B,C)** The reduced brain size of *Mecp2^−/y^* mice affects both, total hemispherical as well as hippocampal areas, and it is not rescued by Trolox. Bar shading and patterns are identical for all panels, the number of slices analyzed also applies to panel **(D)**. **(D)** Cortical layer thickness was assessed in the primary somatosensory cortex as depicted here (arrow mark) in a slice of a PBS-treated WT mouse. Independent of the treatment received, neocortical thinning was still present in all *Mecp2^−/y^* mice. **(E)** The reduced size of MeCP2-deficient CA1 pyramidal neurons, plotted here as soma area, persists upon Trolox treatment. The number of cells analyzed is indicated below each bar. The sample image shows a CA1 *st. pyramidale* segment of a PBS-treated WT mouse. Crosshatches indicate differences between the respective *Mecp2^−/y^* and WT groups (^##^*P* < 0.01, ^###^*P* < 0.001).

MeCP2-deficiency is associated with decreased brain size, thinning of neocortical layers and smaller neurons (Belichenko et al., 2008; Fischer et al., 2009). To screen for changes in these parameters, we performed morphological analyses on perfusion-fixed brains of the treated mice. Nissl staining of thin coronal sections (30 μm) revealed the expected reduction in total brain size in *Mecp2^−/y^* mice (Figure 9A). As referred to PBS-treated WTs, the total hemisphere size in the respective *Mecp2^−/y^* group was smaller by an average of 9.4%, the hippocampal formation was reduced by 7.0% and neocortical layers—as analyzed here for the primary somatosensory cortex (trunk region)—were thinned by 9.1% (Figures 9B–D). The soma of CA1 pyramidal neurons was also smaller in PBS-treated MeCP2-deficient mice by an average of 8.7% than in the respective WTs (Figure 9E). Systemic Trolox treatment did not mediate any effects on these morphological parameters in WT and *Mecp2^−/y^* mice, nor did it reverse the genotypic differences.

## Discussion

Our earlier *in vitro* tests revealed promising effects of Trolox on hippocampal network function in symptomatic *Mecp2^−/y^* mice (Janc and Müller, 2014). As the next logical step we therefore assessed, whether *in vivo* treatment with this free-radical scavenger would be equally efficient and improve the physical constitution of *Mecp2^−/y^* mice or slow down disease progression. Chronic treatment and frequent injections were largely well-tolerated over the entire ~6 weeks. Only a small number of mice (3 WTs, 14 *Mecp2^−/y^*) died or had to be euthanized before the end of treatment. The survival rate of all treated *Mecp2^−/y^* mice was 86% at PD47–50, and this average does not noticeably differ from that of naïve, untreated animals in our Rett mouse colony or the observations of others (Guy et al., 2001). Taking a closer look at the specific survival rates of the different treatment groups (Table 1) indicates, however, that the mice which died or had to be euthanized even included three WTs and that they all (with the exception of one *Mecp2^−/y^* mouse) had received Trolox and not placebo. It therefore seems that even though the administered doses of Trolox were far below the LD50 lethal dose for mice (i.p. injection 1700 mg/kg see http://www.drugfuture.com/toxic/q28-q963.html), some adverse effects of systemic Trolox treatment cannot be excluded.

For example, WT mice receiving high-dose Trolox showed lowered blood glucose levels, and a trend towards decreased motor activity as well as reduced environmental exploration, whereas low-dose Trolox treated WTs had lower FAD/NADH baseline ratios and a tendency to decreased fEPSP amplitudes. The very underlying causes are unclear, but nevertheless this observation emphasizes the importance of proper antioxidant dosing and optimized titration of redox conditions. Without question, cellular viability suffers from an increased oxidant load, but in view of the numerous functions controlled by redox conditions, also an “over-buffering”, i.e., too reducing conditions may be equally damaging.

This preclinical trial is part of a long-lasting research line, and we have reference data on some of the parameters assessed, which were recorded earlier also from naïve mice. Comparing the data sets shows that some of the genotypic differences seen in naïve mice were either not present or less pronounced among PBS-treated WT and *Mecp2^−/y^* mice (Table 3). In several parameters WTs became worse, which dampened the phenotypic differences to *Mecp2^−/y^* mice. An obvious cause may be the frequent injections and animal handling, which might have resulted in adverse stress. During study design, we chose i.p. injections, as they ensure a highly reliable drug delivery and are applicable also to neonates. Osmotic mini pumps are not feasible for mice at the early postnatal age and oral feeding (via drinking water or chow) could only start after weaning, i.e., not before PD20, unless the (foster) mothers are treated as well.

There are of course further reasons which may explain the reduced efficacy of Trolox *in vivo* as compared to *in vitro*. Even though Trolox shows a relatively high blood-brain barrier transferability (Wada et al., 2016), it reaches an antioxidative effect of only ~65% of that observed in blood. Thus, it may not have reached sufficiently high doses to reverse all of these symptoms arising as a consequence of redox-imbalance and oxidative stress. Since higher doses of Trolox cannot be achieved without the use of additional solvents, shorter intervals and/or different routes of administration may be preferable for future trials. Furthermore, recent studies have pointed out that combinations of antioxidants—even applied at lower doses—may potentiate each others efficacy, and that this strategy may be more promising than just increasing the dose of a single compound (Tarnopolsky, 2008; López-Erauskin et al., 2011).

### General Physical Appearance and Blood Parameters

The detailed assessment of body weights and BMI revealed that Trolox treatment did not ameliorate the reduced growth of *Mecp2^−/y^* mice. Once separated from their foster mothers and required to feed on their own, all *Mecp2^−/y^* groups gained less weight than their WT littermates, as was reported earlier also for non-treated *Mecp2^−/y^* mice (Guy et al., 2001).

Weaning marks a switch in metabolism from milk (high fat diet) to mouse chow (carbohydrate rich diet; Ferré et al., 1986), and this transition seems problematic in *Mecp2^−/y^* mice. Loss of MeCP2 function increases insulin levels, decreases insulin signaling and results in a dissociation of insulin levels and their appropriate metabolic effects on glucose regulation (Pitcher et al., 2013; Krishnan et al., 2015). Also in Rett patients, increased glucose utilization indicates altered glucose regulation and/or metabolism (Villemagne et al., 2002). We found ~29% lower blood glucose levels in PBS-treated *Mecp2^−/y^* mice than in WTs. This difference was abolished in *Mecp2^−/y^* mice receiving high or low Trolox doses, indicating that chronic Trolox treatment rescued the altered glucose regulation/metabolism.

Interestingly, it was shown in rats that glucose-mediated production of mitochondrial ROS is mandatory for insulin secretion; higher mitochondrial ROS levels increased insulin secretion, whereas antioxidants decreased it (Leloup et al., 2009). Thus the lower glucose levels observed in placebo treated *Mecp2^−/y^ mice* may at least in part be a consequence of the systemic oxidative stress in these mice, which is then antagonized by Trolox. In contrast in WT mice, high-dose Trolox slightly lowered blood glucose levels by an as yet unidentified mechanism. For male C57BL6 mice (PD 56–70), the vendor specifies a blood glucose content of 13.7 ± 3.4 mM (*n* = 114; see http://www.criver.com/files/pdfs/rms/c57bl6/rm_rm_d_c57bl6n_mouse.aspx). High-dose Trolox treated WTs showed glucose levels of 12.1 ± 2.1 mM (*n* = 23), which are well within this range. As neither the growth curves of high-dose Trolox treated WT mice, nor any other of their behavioral parameters showed a significant decline, adverse systemic consequences of these moderate blood glucose changes seem unlikely.

The hematocrit was not affected by Trolox, and remained increased in all *Mecp2*^−*/y*^ groups. As the high hematocrit represents an adaptive response of *Mecp2*^−*/y*^ mice to irregular breathing and the associated intermittent systemic hypoxia (Stettner et al., 2008; Fischer et al., 2009), this suggests that *Mecp2*^−*/y*^ mice, despite chronic Trolox treatment, still suffered from intermittent systemic hypoxia. The unchanged breathing irregularities detected in all *Mecp2^−/y^* groups during plethysmography support this notion.

### Behavioral Testing and Plethysmography

Motor problems and insufficient motor skills are evident in various MeCP2-mutant mice (Guy et al., 2001; Shahbazian et al., 2002; Moretti et al., 2005; Wegener et al., 2014). Compared to WTs, the treated *Mecp2^−/y^* mice failed markedly earlier on the rotarod, and their impaired motor function was not ameliorated by Trolox. The poor rotarod performance likely arises from an insufficient motor coordination, as is suggested by the pronounced hind limb clasping in symptomatic *Mecp2^−/y^* mice (Guy et al., 2001). General muscle weakness is a less likely cause. We found most *Mecp2^−/y^* mice being able to climb around in their cages, and others confirmed normal muscle strength of *Mecp2^−/y^* mice in wire suspension tests (Santos et al., 2007).

*Mecp2^−/y^* mice are also less active (Guy et al., 2001), which in part may be due to their motor deficits. They traveled shorter distances than WTs, which in the open field and hole board tests was accompanied by a decline in mean velocity and prolonged resting times. Marked genotypic differences in the relative times spent in the defined areas were not found, but low-dose Trolox-treated *Mecp2^−/y^* mice stayed slightly more in the periphery and less in the corners than the respective WTs. Environmental exploration in the hole board was also less pronounced in *Mecp2^−/y^* mice. Yet, the number of head-dips was increased in high-dose Trolox-treated *Mecp2^−/y^* mice as compared to the other *Mecp2^−/y^* groups, and they did no longer differ from high-dose Trolox-treated WTs. This was paralleled by a shortened mean resting time of the respective *Mecp2^−/y^* mice. Accordingly, environmental exploration in *Mecp2^−/y^* mice slightly improved upon Trolox treatment.

One of the most prominent symptoms in RTT is erratic breathing (Julu et al., 2001; Weese-Mayer et al., 2006; Stettner et al., 2008; Katz et al., 2009). Each episode of irregular breathing evokes a drop in arterial O_2_ saturation, which is restored only after normal ventilation resumes. Such intermittent systemic hypoxia may provoke damage and/or induce redox stress in highly vulnerable neuronal tissue. Also the regulation of breathing itself might be affected. H_2_O_2_-evoked oxidative stress modulates the *in vitro* breathing pattern generated in the isolated medullary pre-Bötzinger Complex of neonatal WT mice: Biphasic responses were reported, consisting of an initial depression followed by augmented respiratory activity (Garcia et al., 2011). Therefore, we also assessed whether Trolox may improve breathing. As expected, *Mecp2^−/y^* mice showed highly irregular breathing patterns. High- or low-dose Trolox did not improve the regularity of breathing, and the number and duration of apneas did not decrease. Yet, a further deterioration of breathing was not observed either. It should be mentioned though that the individual *Mecp2^−/y^* mice varied greatly; some displayed only mild breathing disturbances with just 1–2 apneas during the evaluated 3 min interval, whereas others showed up to 49 apneas exceeding 750 ms.

### Hypoxia Susceptibility and Synaptic Function

In contrast to our earlier studies on naïve mice (Fischer et al., 2009; Janc and Müller, 2014), PBS-treated WT and *Mecp2^−/y^* mice did not differ in their hypoxia susceptibility assessed as time to HSD onset. Nevertheless, treatment with low-dose Trolox increased the hypoxia tolerance of *Mecp2^−/y^* mice as compared to the respective WT and the PBS-treated *Mecp2^−/y^* group. This effect was not seen with high-dose Trolox, emphasizing the importance of well-adjusted cellular redox balance and careful antioxidant dosing. In view of the irregular breathing and intermittent hypoxia, the Trolox-mediated increase in hypoxia tolerance is clearly of merit, as it may prevent additional complications especially in highly anoxia-vulnerable neuronal networks such as the hippocampus and cortex.

Synaptic plasticity is impaired in *Mecp2*-mutant mice (Asaka et al., 2006; Moretti et al., 2006; Fischer et al., 2009; Janc and Müller, 2014). Yet, the differences among PBS-treated *Mecp2^−/y^* and WT mice were less evident than in naïve mice (Janc and Müller, 2014). As suggested by the normalized fEPSP amplitudes recorded in CA1 *st. radiatum* (Table 3), this seems to result from PBS-treated WTs showing a higher degree of neuronal excitability than naïve WT mice. This variability of genotypic differences in synaptic plasticity may be explained by earlier reports identifying stress and stress-hormones as a cause for impaired hippocampal LTP (Kim and Yoon, 1998; Kim and Diamond, 2002). Repeated i.p. injections for several weeks could represent a chronic stressor (Ryabinin et al., 1999) which induces stress hormone release and dampens LTP in slices of frequently handled/injected WT mice. In *Mecp2^−/y^* mice, LTP is already impaired to a degree that it may not have responded to any additional stress.

Nevertheless, *in vivo* Trolox treatment mediated some positive effects on synaptic plasticity. Low-dose Trolox-treated *Mecp2^−/y^* mice showed a significantly improved STP as compared to the respective WTs and the other *Mecp2^−/y^* groups. The final amount of LTP also tended to increase, but did not reach the level of significance. High-dose Trolox treatment failed to improve synaptic plasticity in *Mecp2^−/y^* or WT mice. This may result from a probably too high concentration mediating adverse effects. As a certain ROS level is necessary for signal transduction cascades during normal physiological processes (Serrano and Klann, 2004), optimum synaptic plasticity can be achieved in a well-balanced redox environment only. Accordingly, superoxide scavenging and overexpression of superoxide dismutase 3 impair hippocampal LTP (Klann et al., 1998; Thiels et al., 2000). Along this line, high-dose Trolox may have shifted hippocampal redox balance beyond the optimum. Similar effects we found earlier in naïve mice, in which Trolox rescued STP and LTP in *Mecp2^−/y^* hippocampal slices, but dampened it in WTs (Janc and Müller, 2014).

### Mitochondrial Metabolism and Function

Trolox did not mediate any consistent changes in mitochondrial metabolism or polarization. A slight decrease of the FAD/NADH baseline ratio was found in low-dose Trolox treated WTs, but this was not accompanied by any changes in mitochondrial polarization, estimated here as Rh123 fluorescence. Hence, general negative side effects of this compound on mitochondrial function can be excluded. In contrast to our earlier studies on naïve mice (Großer et al., 2012; Janc and Müller, 2014), PBS injected/handled WT and *Mecp2^−/y^* did no longer differ in their hippocampal FAD/NADH ratios and Rh123 responses (Table 3). Again, the stress associated with the frequent i.p. injections, might have affected mitochondrial metabolism, thereby diminishing the genotypic differences. Most primary mediators of stress responses exert numerous effects on mitochondrial metabolism and ROS generation and may even induce apoptosis (Manoli et al., 2007).

### Lipid Peroxidation and Protein Carbonylation

RTT is closely linked to oxidative stress and redox imbalance (Sierra et al., 2001; De Felice et al., 2011, 2012b; Großer et al., 2012; Müller and Can, 2014). Unclear is, however, which particular brain regions are affected and whether the oxidative damage mainly targets proteins, lipids or both. Earlier studies on whole brains of various Rett mouse models indicated that in particular lipids are subject to oxidative damage, whereas protein damage is not detectable in each model (De Felice et al., 2014). Our comparison of neocortical tissue of PBS-treated WT and *Mecp2^−/y^* mice did not reveal obviously different levels of lipid peroxidation, but high-dose Trolox decreased the extent of lipid peroxidation in *Mecp2^−/y^* neocortex below WT levels. Furthermore, the increased protein carbonyl content detected in PBS treated *Mecp2^−/y^* mice, was no longer evident upon Trolox treatment. This confirms that systemic Trolox administration is capable of ameliorating oxidative brain tissue damage. However, it also should be noted here that the sensitivity range of these assays may not be ideal to detect reliably also more subtle changes in protein and lipid oxidation. We verified both assays by provoking oxidative tissue damage via direct oxidant (TBHP) treatment. This resulted in a clear peroxidation of lipids and carbonylation of proteins in neocortical samples, but it also revealed that despite direct oxidant stress only marked but not massive changes in spectrophotometric readout were obtained.

### Merits of Antioxidant and Free-Radical Scavenger Treatment in RTT

Various earlier studies verified that antioxidants and free-radical scavengers ameliorate certain aspects of the complex clinical appearance of RTT (Chapleau et al., 2013). In female Rett mice, alterations in vascular endothelium were reversed by administration of curcumin, an anti-inflammatory compound which also mediates antioxidative effects (Panighini et al., 2013). In Rett patients, ω-3 PUFA-supplementation led to an improvement of motor function, non-verbal communication and breathing regularity (De Felice et al., 2012a; Maffei et al., 2014). Ultimate proof for a pathogenic role of oxidative alterations in RTT is the brain-specific reactivation of the *Mecp2* gene, which resulted in a clear phenotypic improvement in concert with a normalization of various oxidative stress markers (De Felice et al., 2014).

As vitamin E levels are decreased in blood serum of Rett patients (Formichi et al., 1998) and as this vitamin as well as its derivatives act as chain-breaking antioxidants, which prevent the propagation of free radicals in membranes and plasma lipoproteins (Traber and Stevens, 2011; Alberto et al., 2013), these compounds appear especially promising. Applied *in vivo* by oral uptake or i.p. injection, Trolox ameliorates ischemia-induced hippocampal cell loss and 3-NPA-mediated striatal lesions (Gupta and Sharma, 2006; Al Mutairy et al., 2010) as well as oxidative-stress related retinal damage (Dorfman et al., 2006). Our previous *in vitro* tests confirmed the efficacy of Trolox also in a Rett mouse model, in which it improved synaptic function as well as hypoxia tolerance and opposed neuronal hyperexcitability in *Mecp2^−/y^* hippocampal slices (Janc and Müller, 2014).

In the present study, the systemic administration of Trolox improved some of the tested parameters as well. Yet, this *in vivo* Trolox treatment did not noticeably change the most characteristic Rett symptoms. Obvious signs that the disease progression was slowed down in *Mecp2^−/y^* mice were not obtained either. It has to be considered though that these male Rett mice are very severely affected. Therefore, to understand completely the merits of systemic Trolox administration, it needs to be assessed in future trials, how efficient this treatment would be in heterozygous female Rett mice. They represent the conditions of a cellular MeCP2 mosaicism, truthfully reflecting the genetic conditions of the female patients. As disease progression and symptom severity are milder in female Rett (*Mecp2^+/−^*) mice, this will require, however, prolonged treatment durations of several months or even over a year. This certainly cannot be achieved by i.p. injections and the associated stress, as we learned in this present study.

In conclusion, this detailed *in vivo* study confirms some pharmacotherapeutic merit of systemically applied antioxidants in MeCP2-deficient brains. Unfortunately, frequent animal handling and repeated injections apparently modulated the phenotypic appearance of mice and diminished the genotype-related differences of numerous parameters. Therefore, the route of drug administration and the frequent mouse handling are critical issues in study design, which need to be further optimized in future trials. Also, as Trolox ameliorated only a few of the multiple complex Rett symptoms, one may consider its use as adjuvant, i.e., its combination with other compounds improving different symptoms, in order to complement the spectrum of effects. For example, respiration, which was not improved by Trolox was shown earlier to be normalized by norepinephrine uptake blockers, GABA uptake inhibitors, and serotonin 1a receptor agonists (Zanella et al., 2008; Abdala et al., 2010) or the antidepressant mirtazapine which furthermore prevented cortical atrophy (Bittolo et al., 2016). In view of the complex phenotype of RTT, such combination therapies, designed to the particular needs of each individual patient are probably the most promising but also the most challenging strategy.

## Author Contributions

OAJ: planned and conducted experiments, performed animal treatment, analyzed data and contributed to manuscript writing. MAH: planned and conducted experiments, analyzed data, prepared figures. KD: analyzed data, prepared figures, contributed to manuscript writing. BK: conducted experiments, performed animal treatment, contributed to data analysis and genotyped animals. CM: performed animal treatment and planned experiments. SH: planned experiments, analyzed data and contributed to manuscript writing. MM: planned and supervised the study, coordinated projects and finalized the manuscript. All authors read and approved the final version of the manuscript.

## Conflict of Interest Statement

The authors declare that the research was conducted in the absence of any commercial or financial relationships that could be construed as a potential conflict of interest.

## References

[B1] Abdala A. P. Dutschmann M. Bissonnette J. M. Paton J. F. (2010). Correction of respiratory disorders in a mouse model of Rett syndrome. Proc. Natl. Acad. Sci. U S A 107, 18208–18213. 10.1073/pnas.1012104107 20921395 PMC2964253

[B2] Aizenman E. Lipton S. A. Loring R. H. (1989). Selective modulation of NMDA responses by reduction and oxidation. Neuron 2, 1257–1263. 10.1016/0896-6273(89)90310-3 2696504

[B4] Alberto M. E. Russo N. Grand A. Galano A. (2013). A physicochemical examination of the free radical scavenging activity of Trolox: mechanism, kinetics and influence of the environment. Phys. Chem. Chem. Phys. 15, 4642–4650. 10.1039/c3cp43319f 23423333

[B3] Al Mutairy A. Al Kadasah S. Elfaki I. Arshaduddin M. Malik D. Al Moutaery K.. (2010). Trolox ameliorates 3-nitropropionic acid-induced neurotoxicity in rats. Neurotoxicol. Teratol. 32, 226–233. 10.1016/j.ntt.2009.09.003 19755148

[B5] Amir R. E. Van den Veyver I. B. Wan M. Tran C. Q. Francke U. Zoghbi H. Y. (1999). Rett syndrome is caused by mutations in X-linked MECP2, encoding methyl-CpG-binding protein 2. Nat. Genet. 23, 185–188. 10.1038/13810 10508514

[B6] Asaka Y. Jugloff D. G. M. Zhang L. Eubanks J. H. Fitzsimonds R. M. (2006). Hippocampal synaptic plasticity is impaired in the Mecp2-null mouse model of Rett syndrome. Neurobiol. Dis. 21, 217–227. 10.1016/j.nbd.2005.07.005 16087343

[B7] Barthe J. Y. Clarac F. (1997). Modulation of the spinal network for locomotion by substance P in the neonatal rat. Exp. Brain Res. 115, 485–492. 10.1007/pl00005718 9262203

[B8] Baxter L. L. Marugan J. J. Xiao J. Incao A. Mckew J. C. Zheng W.. (2012). Plasma and tissue concentrations of α-tocopherol and δ-tocopherol following high dose dietary supplementation in mice. Nutrients 4, 467–490. 10.3390/nu4060467 22822447 PMC3397347

[B9] Belichenko N. P. Belichenko P. V. Li H. H. Mobley W. C. Francke U. (2008). Comparative study of brain morphology in Mecp2 mutant mouse models of Rett syndrome. J. Comp. Neurol. 508, 184–195. 10.1002/cne.21673 18306326

[B10] Belichenko P. V. Wright E. E. Belichenko N. P. Masliah E. Li H. H. Mobley W. C.. (2009). Widespread changes in dendritic and axonal morphology in Mecp2-mutant mouse models of Rett syndrome: evidence for disruption of neuronal networks. J. Comp. Neurol. 514, 240–258. 10.1002/cne.22009 19296534

[B11] Bittolo T. Raminelli C. A. Deiana C. Baj G. Vaghi V. Ferrazzo S.. (2016). Pharmacological treatment with mirtazapine rescues cortical atrophy and respiratory deficits in MeCP2 null mice. Sci. Rep. 6:19796. 10.1038/srep19796 26806603 PMC4726391

[B12] Blue M. E. Naidu S. Johnston M. V. (1999). Altered development of glutamate and GABA receptors in the basal ganglia of girls with Rett syndrome. Exp. Neurol. 156, 345–352. 10.1006/exnr.1999.7030 10328941

[B13] Calfa G. Hablitz J. J. Pozzo-Miller L. (2011). Network hyperexcitability in hippocampal slices from Mecp2 mutant mice revealed by voltage-sensitive dye imaging. J. Neurophysiol. 105, 1768–1784. 10.1152/jn.00800.2010 21307327 PMC3075283

[B14] Chahrour M. Zoghbi H. Y. (2007). The story of Rett syndrome: from clinic to neurobiology. Neuron 56, 422–437. 10.1016/j.neuron.2007.10.001 17988628

[B15] Chapleau C. A. Lane J. Pozzo-Miller L. Percy A. K. (2013). Evaluation of current pharmacological treatment options in the management of Rett syndrome: from the present to future therapeutic alternatives. Curr. Clin. Pharmacol. 8, 358–369. 10.2174/15748847113086660069 24050745 PMC3789853

[B16] Dani V. S. Chang Q. Maffei A. Turrigiano G. G. Jaenisch R. Nelson S. B. (2005). Reduced cortical activity due to a shift in the balance between excitation and inhibition in a mouse model of Rett syndrome. Proc. Natl. Acad. Sci. U S A 102, 12560–12565. 10.1073/pnas.0506071102 16116096 PMC1194957

[B17] De Felice C. Ciccoli L. Leoncini S. Signorini C. Rossi M. Vannuccini L.. (2009). Systemic oxidative stress in classic Rett syndrome. Free Radic. Biol. Med. 47, 440–448. 10.1016/j.freeradbiomed.2009.05.016 19464363

[B18] De Felice C. Della Ragione F. Signorini C. Leoncini S. Pecorelli A. Ciccoli L.. (2014). Oxidative brain damage in Mecp2-mutant murine models of Rett syndrome. Neurobiol. Dis. 68, 66–77. 10.1016/j.nbd.2014.04.006 24769161 PMC4076513

[B19] De Felice C. Signorini C. Durand T. Ciccoli L. Leoncini S. D’Esposito M.. (2012a). Partial rescue of Rett syndrome by ω-3 polyunsaturated fatty acids (PUFAs) oil. Genes Nutr. 7, 447–458. 10.1007/s12263-012-0285-7 22399313 PMC3380188

[B21] De Felice C. Signorini C. Leoncini S. Pecorelli A. Durand T. Valacchi G.. (2012b). The role of oxidative stress in Rett syndrome: an overview. Ann. N Y Acad. Sci. 1259, 121–135. 10.1111/j.1749-6632.2012.06611.x 22758644

[B20] De Felice C. Signorini C. Durand T. Oger C. Guy A. Bultel-Poncé V.. (2011). F2-dihomo-isoprostanes as potential early biomarkers of lipid oxidative damage in Rett syndrome. J. Lipid Res. 52, 2287–2297. 10.1194/jlr.p017798 21917727 PMC3283260

[B22] De Filippis B. Valenti D. de Bari L. De Rasmo D. Musto M. Fabbri A.. (2015). Mitochondrial free radical overproduction due to respiratory chain impairment in the brain of a mouse model of Rett syndrome: protective effect of CNF1. Free Radic. Biol. Med. 83, 167–177. 10.1016/j.freeradbiomed.2015.02.014 25708779

[B23] Dorfman A. L. Dembinska O. Chemtob S. Lachapelle P. (2006). Structural and functional consequences of trolox C treatment in the rat model of postnatal hyperoxia. Invest. Ophthalmol. Vis. Sci. 47, 1101–1108. 10.1167/iovs.05-0727 16505047

[B24] Dotti M. T. Manneschi L. Malandrini A. De Stefano N. Caznerale F. Federico A. (1993). Mitochondrial dysfunction in Rett syndrome. An ultrastructural and biochemical study. Brain Dev. 15, 103–106. 10.1016/0387-7604(93)90045-a 8214327

[B25] Duchen M. R. Biscoe T. J. (1992). Mitochondrial function in type I cells isolated from rabbit arterial chemoreceptors. J. Physiol. 450, 13–31. 10.1113/jphysiol.1992.sp019114 1432706 PMC1176109

[B26] Dudchenko P. A. (2004). An overview of the tasks used to test working memory in rodents. Neurosci. Biobehav. Rev. 28, 699–709. 10.1016/j.neubiorev.2004.09.002 15555679

[B27] Eeg-Olofsson O. Al-Zuhair A. G. Teebi A. S. Daoud A. S. Zaki M. Besisso M. S.. (1990). Rett syndrome: a mitochondrial disease? J. Child Neurol. 5, 210–214. 10.1177/088307389000500311 2168910

[B28] Emaus R. K. Grunwald R. Lemasters J. J. (1986). Rhodamine 123 as a probe of transmembrane potential in isolated rat-liver mitochondria: spectral and metabolic properties. Biochim. Biophys. Acta 850, 436–448. 10.1016/0005-2728(86)90112-x 2873836

[B29] Ferré P. Decaux J. F. Issad T. Girard J. (1986). Changes in energy-metabolism during the suckling and weaning period in the newborn. Reprod. Nutr. Dev. 26, 619–631. 10.1051/rnd:19860413 3523657

[B30] Filosa S. Pecorelli A. D’Esposito M. Valacchi G. Hajek J. (2015). Exploring the possible link between MeCP2 and oxidative stress in Rett syndrome. Free Radic. Biol. Med. 88, 81–90. 10.1016/j.freeradbiomed.2015.04.019 25960047

[B31] Fischer M. Reuter J. Gerich F. J. Hildebrandt B. Hägele S. Katschinski D.. (2009). Enhanced hypoxia susceptibility in hippocampal slices from a mouse model of Rett syndrome. J. Neurophysiol. 101, 1016–1032. 10.1152/jn.91124.2008 19073793

[B32] Formichi P. Battisti C. Dotti M. T. Hayek G. Zappella M. Federico A. (1998). Vitamin E serum levels in Rett syndrome. J. Neurol. Sci. 156, 227–230. 10.1016/s0022-510x(98)00035-5 9588862

[B33] Foster K. A. Galeffi F. Gerich F. J. Turner D. A. Müller M. (2006). Optical and pharmacological tools to investigate the role of mitochondria during oxidative stress and neurodegeneration. Prog. Neurobiol. 79, 136–171. 10.1016/j.pneurobio.2006.07.001 16920246 PMC1994087

[B34] Funke F. Dutschmann M. Müller M. (2007). Imaging of respiratory-related population activity with single-cell resolution. Am. J. Physiol. Cell Physiol. 292, C508–C516. 10.1152/ajpcell.00253.2006 16956966

[B35] Funke F. Gerich F. J. Müller M. (2011). Dynamic, semi-quantitative imaging of intracellular ROS levels and redox status in rat hippocampal neurons. Neuroimage 54, 2590–2602. 10.1016/j.neuroimage.2010.11.031 21081169

[B36] Garcia A. J. III Khan S. A. Kumar G. K. Prabhakar N. R. Ramirez J.-M. (2011). Hydrogen peroxide differentially affects activity in the pre-Bötzinger complex and hippocampus. J. Neurophysiol. 106, 3045–3055. 10.1152/jn.00550.2010 21849609 PMC3234088

[B37] Gerich F. J. Hepp S. Probst I. Müller M. (2006). Mitochondrial inhibition prior to oxygen-withdrawal facilitates the occurrence of hypoxia-induced spreading depression in rat hippocampal slices. J. Neurophysiol. 96, 492–504. 10.1152/jn.01015.2005 16611842

[B38] Großer E. Hirt U. Janc O. A. Menzfeld C. Fischer M. Kempkes B.. (2012). Oxidative burden and mitochondrial dysfunction in a mouse model of Rett syndrome. Neurobiol. Dis. 48, 102–114. 10.1016/j.nbd.2012.06.007 22750529

[B39] Gupta S. Sharma S. S. (2006). Neuroprotective effects of trolox in global cerebral ischemia in gerbils. Biol. Pharm. Bull. 29, 957–961. 10.1248/bpb.29.957 16651726

[B40] Guy J. Hendrich B. Holmes M. Martin J. E. Bird A. (2001). A mouse Mecp2-null mutation causes neurological symptoms that mimic Rett syndrome. Nat. Genet. 27, 322–326. 10.1038/85899 11242117

[B41] Hagberg B. (1985). Rett’s syndrome: prevalence and impact on progressive severe mental retardation in girls. Acta Paediatr. Scand. 74, 405–408. 10.1111/j.1651-2227.1985.tb10993.x 4003065

[B42] Hammarström A. K. M. Gage P. W. (2000). Oxygen-sensing persistent sodium channels in rat hippocampus. J. Physiol. 529, 107–118. 10.1111/j.1469-7793.2000.00107.x 11080255 PMC2270177

[B43] Hanson G. T. Aggeler R. Oglesbee D. Cannon M. Capaldi R. A. Tsien R. Y.. (2004). Investigating mitochondrial redox potential with redox-sensitive green fluorescent protein indicators. J. Biol. Chem. 279, 13044–13053. 10.1074/jbc.M312846200 14722062

[B44] Hepp S. Gerich F. J. Müller M. (2005). Sulfhydryl oxidation reduces hippocampal susceptibility to hypoxia-induced spreading depression by activating BK-channels. J. Neurophysiol. 94, 1091–1103. 10.1152/jn.00291.2005 15872065

[B45] Hidalgo C. Bull R. Behrens M. I. Donoso P. (2004). Redox regulation of RyR-mediated Ca^2+^ release in muscle and neurons. Biol Res. 37, 539–552. 10.4067/s0716-97602004000400007 15709680

[B46] Janc O. A. Müller M. (2014). The free radical scavenger Trolox dampens neuronal hyperexcitability, reinstates synaptic plasticity and improves hypoxia tolerance in a mouse model of Rett syndrome. Front. Cell. Neurosci. 8:56. 10.3389/fncel.2014.00056 24605086 PMC3932407

[B47] Julu P. O. Kerr A. M. Apartopoulos F. Al-Rawas S. Engerström I. W. Engerström L.. (2001). Characterisation of breathing and associated central autonomic dysfunction in the Rett disorder. Arch. Dis. Child. 85, 29–37. 10.1136/adc.85.1.29 11420195 PMC1718860

[B48] Katz D. M. Dutschmann M. Ramirez J. M. Hilaire G. (2009). Breathing disorders in Rett syndrome: progressive neurochemical dysfunction in the respiratory network after birth. Respir. Physiol. Neurobiol. 168, 101–108. 10.1016/j.resp.2009.04.017 19394452 PMC2758855

[B49] Kim J. J. Diamond D. M. (2002). The stressed hippocampus, synaptic plasticity and lost memories. Nat. Rev. Neurosci. 3, 453–462. 10.1038/nrn849 12042880

[B50] Kim J. J. Yoon K. S. (1998). Stress: metaplastic effects in the hippocampus. Trends Neurosci. 21, 505–509. 10.1016/s0166-2236(98)01322-8 9881846

[B51] Klann E. Roberson E. D. Knapp L. T. Sweatt J. D. (1998). A role for superoxide in protein kinase C activation and induction of long-term potentiation. J. Biol. Chem. 273, 4516–4522. 10.1074/JBC.273.8.4516 9468506

[B52] Kriaucionis S. Paterson A. Curtis J. Guy J. Macleod N. Bird A. (2006). Gene expression analysis exposes mitochondrial abnormalities in a mouse model of Rett syndrome. Mol. Cell Biol. 26, 5033–5042. 10.1128/mcb.01665-05 16782889 PMC1489175

[B53] Krishnan N. Krishnan K. Connors C. R. Choy M. S. Page R. Peti W.. (2015). PTP1B inhibition suggests a therapeutic strategy for Rett syndrome. J. Clin. Invest. 125, 3163–3177. 10.1172/JCI80323 26214522 PMC4563751

[B54] Leloup C. Tourrel-Cuzin C. Magnan C. Karaca M. Castel J. Carneiro L.. (2009). Mitochondrial reactive oxygen species are obligatory signals for glucose-induced insulin secretion. Diabetes 58, 673–681. 10.2337/db07-1056 19073765 PMC2646066

[B55] López-Erauskin J. Fourcade S. Galino J. Ruiz M. Schlüter A. Naudi A.. (2011). Antioxidants halt axonal degeneration in a mouse model of X-adrenoleukodystrophy. Ann. Neurol. 70, 84–92. 10.1002/ana.22363 21786300 PMC3229843

[B56] Maffei S. De Felice C. Cannarile P. Leoncini S. Signorini C. Pecorelli A.. (2014). Effects of omega-3 PUFAs supplementation on myocardial function and oxidative stress markers in typical Rett syndrome. Mediators Inflamm. 2014:983178. 10.1155/2014/983178 24526821 PMC3913460

[B57] Manoli I. Alesci S. Blackman M. R. Su Y. A. Rennert O. M. Chrousos G. P. (2007). Mitochondria as key components of the stress response. Trends Endocrinol. Metab. 18, 190–198. 10.1016/j.tem.2007.04.004 17500006

[B58] Medrihan L. Tantalaki E. Aramuni G. Sargsyan V. Dudanova I. Missler M.. (2008). Early defects of GABAergic synapses in the brain stem of a MeCP2 mouse model of Rett syndrome. J. Neurophysiol. 99, 112–121. 10.1152/jn.00826.2007 18032561

[B59] Moretti P. Bouwknecht J. A. Teague R. Paylor R. Zoghbi H. Y. (2005). Abnormalities of social interactions and home-cage behavior in a mouse model of Rett syndrome. Hum. Mol. Genet. 14, 205–220. 10.1093/hmg/ddi016 15548546

[B60] Moretti P. Levenson J. M. Battaglia F. Atkinson R. Teague R. Antalffy B.. (2006). Learning and memory and synaptic plasticity are impaired in a mouse model of Rett syndrome. J. Neurosci. 26, 319–327. 10.1523/JNEUROSCI.2623-05.2006 16399702 PMC6674314

[B62] Müller W. Bittner K. (2002). Differential oxidative modulation of voltage-dependent K^+^ currents in rat hippocampal neurons. J. Neurophysiol. 87, 2990–2995. 10.1152/jn.00790.2001 12037202

[B61] Müller M. Can K. (2014). Aberrant redox homoeostasis and mitochondrial dysfunction in Rett syndrome. Biochem. Soc. Trans. 42, 959–964. 10.1042/BST20140071 25109986

[B63] Panighini A. Duranti E. Santini F. Maffei M. Pizzorusso T. Funel N.. (2013). Vascular dysfunction in a mouse model of Rett syndrome and effects of curcumin treatment. PLoS One 8:e64863. 10.1371/journal.pone.0064863 23705018 PMC3660336

[B64] Pelka G. J. Watson C. M. Radziewic T. Hayward M. Lahooti H. Christodoulou J.. (2006). *Mecp2* deficiency is associated with learning and cognitive deficits and altered gene activity in the hippocampal region of mice. Brain 129, 887–898. 10.1093/brain/awl022 16467389

[B65] Pitcher M. R. Ward C. S. Arvide E. M. Chapleau C. A. Pozzo-Miller L. Hoeflich A.. (2013). Insulinotropic treatments exacerbate metabolic syndrome in mice lacking MeCP2 function. Hum. Mol. Genet. 22, 2626–2633. 10.1093/hmg/ddt111 23462290 PMC3674803

[B66] Rett A. (1966). Über ein eigenartiges hirnatrophisches Syndrom bei Hyperammonämie im Kindesalter. Wien. Med. Wochenschr. 116, 723–726. 5300597

[B67] Ryabinin A. E. Wang Y. M. Finn D. A. (1999). Different levels of Fos immunoreactivity after repeated handling and injection stress in two inbred strains of mice. Pharmacol. Biochem. Behav. 63, 143–151. 10.1016/s0091-3057(98)00239-1 10340535

[B68] Sah R. Galeffi F. Ahrens R. Jordan G. Schwartz-Bloom R. D. (2002). Modulation of the GABA_A_-gated chloride channel by reactive oxygen species. J. Neurochem. 80, 383–391. 10.1046/j.0022-3042.2001.00706.x 11905987

[B69] Santos M. Silva-Fernandes A. Oliveira P. Sousa N. Maciel P. (2007). Evidence for abnormal early development in a mouse model of Rett syndrome. Genes Brain Behav. 6, 277–286. 10.1111/j.1601-183x.2006.00258.x 16848781

[B70] Serrano F. Klann E. (2004). Reactive oxygen species and synaptic plasticity in the aging hippocampus. Ageing Res. Rev. 3, 431–443. 10.1016/j.arr.2004.05.002 15541710

[B71] Shahbazian M. Young J. Yuva-Paylor L. Spencer C. Antalffy B. Noebels J.. (2002). Mice with truncated MeCP2 recapitulate many Rett syndrome features and display hyperacetylation of histone H3. Neuron 35, 243–254. 10.1016/s0896-6273(02)00768-7 12160743

[B72] Shahbazian M. D. Zoghbi H. Y. (2001). Molecular genetics of Rett syndrome and clinical spectrum of MECP2 mutations. Curr. Opin. Neurol. 14, 171–176. 10.1097/00019052-200104000-00006 11262731

[B73] Sierra C. Vilaseca M. A. Brandi N. Artuch R. Mira A. Nieto M.. (2001). Oxidative stress in Rett syndrome. Brain Dev. 23, S236–S239. 10.1016/s0387-7604(01)00369-2 11738881

[B74] Signorini C. De Felice C. Leoncini S. Møller R. S. Zollo G. Buoni S.. (2016). MECP2 duplication syndrome: evidence of enhanced oxidative stress. A comparison with Rett syndrome. PLoS One 11:e0150101. 10.1371/journal.pone.0150101 26930212 PMC4773238

[B75] Stettner G. M. Huppke P. Gärtner J. Richter D. W. Dutschmann M. (2008). Disturbances of breathing in Rett syndrome: results from patients and animal models. Adv. Exp. Med. Biol. 605, 503–507. 10.1007/978-0-387-73693-8_88 18085325

[B76] Tarnopolsky M. A. (2008). The mitochondrial cocktail: rationale for combined nutraceutical therapy in mitochondrial cytopathies. Adv. Drug Deliv. Rev. 60, 1561–1567. 10.1016/j.addr.2008.05.001 18647623

[B77] Telgkamp P. Cao Y. Q. Basbaum A. I. Ramirez J.-M. (2002). Long-term deprivation of substance P in PPT-A mutant mice alters the anoxic response of the isolated respiratory network. J. Neurophysiol. 88, 206–213 10.1152/jn.00676.2001 12091546

[B78] Thiels E. Urban N. N. Gonzalez-Burgos G. R. Kanterewicz B. I. Barrionuevo G. Chu C. T.. (2000). Impairment of long-term potentiation and associative memory in mice that overexpress extracellular superoxide dismutase. J. Neurosci. 20, 7631–7639. 11027223 10.1523/JNEUROSCI.20-20-07631.2000 PMC6772863

[B79] Traber M. G. Stevens J. F. (2011). Vitamins C and E: beneficial effects from a mechanistic perspective. Free Radic. Biol. Med. 51, 1000–1013. 10.1016/j.freeradbiomed.2011.05.017 21664268 PMC3156342

[B80] Villemagne P. M. Naidu S. Villemagne V. L. Yaster M. Wagner H. N. Jr. Harris J. C.. (2002). Brain glucose metabolism in Rett syndrome. Pediatr. Neurol. 27, 117–122. 10.1016/s0887-8994(02)00399-5 12213612

[B81] Wada M. Wada M. Ikeda R. Fuchigami Y. Koyama H. Ohkawara S.. (2016). Quantitative and antioxidative behavior of Trolox in rats’ blood and brain by HPLC-UV and SMFIA-CL methods. Luminescence 31, 414–418. 10.1002/bio.2975 26192550

[B82] Weese-Mayer D. E. Lieske S. P. Boothby C. M. Kenny A. S. Bennett H. L. Silvestri J. M.. (2006). Autonomic nervous system dysregulation: breathing and heart rate perturbation during wakefulness in young girls with Rett syndrome. Pediatr. Res. 60, 443–449. 10.1203/01.pdr.0000238302.84552.d0 16940240

[B83] Wegener E. Brendel C. Fischer A. Hülsmann S. Gärtner J. Huppke P. (2014). Characterization of the MeCP2^R168X^ knockin mouse model for Rett syndrome. PLoS One 9:e115444. 10.1371/journal.pone.0115444 25541993 PMC4277341

[B84] Zanella S. Mebarek S. Lajard A. M. Picard N. Dutschmann M. Hilaire G. (2008). Oral treatment with desipramine improves breathing and life span in Rett syndrome mouse model. Respir. Physiol. Neurobiol. 160, 116–121. 10.1016/j.resp.2007.08.009 17905670

[B85] Zhang L. He J. Jugloff D. G. Eubanks J. H. (2008). The MeCP2-null mouse hippocampus displays altered basal inhibitory rhythms and is prone to hyperexcitability. Hippocampus 18, 294–309. 10.1002/hipo.20389 18058824

